# Comparative clinical efficacy and safety of perioperative systemic glucocorticoids in primary unilateral total hip arthroplasty: a GRADE-assessed meta-analysis of randomized controlled trials

**DOI:** 10.1186/s42836-026-00397-4

**Published:** 2026-06-02

**Authors:** Omar Abdelaziz, Ziad G. Zayed, Mohamed Abdo Khalafallah, Ahmed Mohamed Elsayed, Islam Saeed Elhois, Khaled A. Elmenawi

**Affiliations:** 1 Faculty of Medicine, Alexandria University, Alexandria, 21526 Egypt; 2 Faculty of Medicine, Al-Azhar University, New Damietta, 34517 Egypt; 3 Arthroplasty Unit, Department of Orthopaedics, Qena General Hospital, Ministry of Health and Population, Qena, 83511 Egypt; 4 Department of Orthopaedic Surgery, Cleveland Clinic, Cleveland, OH 44195 USA

**Keywords:** Total hip arthroplasty, Glucocorticoids, Postoperative pain, Postoperative nausea and vomiting (PONV), Enhanced Recovery after Surgery (ERAS), Meta-analysis

## Abstract

**Background:**

Recovery from primary unilateral total hip arthroplasty (THA) is frequently impeded by pain and postoperative nausea and vomiting (PONV), which hinder Enhanced Recovery After Surgery (ERAS) goals. Although perioperative glucocorticoids offer a multimodal strategy to address these challenges, evidence remains inconsistent, and prior syntheses have often combined THA with total knee arthroplasty, preventing definitive conclusions. Therefore, we conducted this GRADE-assessed meta-analysis to determine the specific efficacy and safety of systemic glucocorticoids in primary unilateral THA.

**Methods:**

We conducted a comprehensive search of PubMed, Web of Science, Scopus, and the Cochrane Library for randomized controlled trials (RCTs) published through October 17, 2025. Twelve RCTs met the inclusion criteria. Primary outcomes included pain (Visual Analog Scale, VAS), PONV incidence, opioid consumption, and glycemic control. Secondary outcomes included length of stay (LOS), C-reactive protein (CRP), interleukin-6 (IL-6), and complications. Risk of bias was assessed using the Cochrane RoB 2 tool. Data were pooled using a random-effects model, and results were expressed as mean differences (MDs) or odds ratios (ORs) with 95% confidence intervals (CIs).

**Results:**

A total of 1,128 patients were included in this meta-analysis. The pooled analysis revealed that systemic glucocorticoids were associated with a significant reduction in pain at rest (MD − 0.20 cm; 95% CI − 0.38 to − 0.01; *p* = 0.04; I^2^ = 58.80%) and during walking (MD − 0.50; 95% CI − 0.89 to − 0.12; *p* = 0.01; I^2^ = 86.06%). All observed pain reductions, including the most notable on postoperative day 1 (POD1) for walking pain (MD − 1.19 cm), were below the THA-specific minimally clinically important difference (MCID) threshold of − 1.86 cm, derived from acute postoperative pain measurements during the hospital stay (POD0–3). No differences were observed in postoperative glucose levels (MD − 0.01 mmol/L; 95% CI − 0.15 to 0.13; *p* = 0.84; I^2^ = 0.00%). Glucocorticoids showed reductions in VAS nausea severity (MD − 0.68; 95% CI − 0.81 to − 0.56; *p* < 0.001; I^2^ = 0.00%), PONV incidence (OR 0.21; 95% CI 0.13–0.35; *p* < 0.001; I^2^ = 0.00%), and rescue antiemetic use (OR 0.33; 95% CI 0.20–0.54; *p* < 0.001; I^2^ = 0.00%). An increase in flexion range of motion was observed (MD 6.82 degrees; 95% CI 2.12–11.52; *p* < 0.001; I^2^ = 94.05%), although this was based on a small number of trials with high heterogeneity. Within the studied timeframe and patient populations, no signal of increased harm was detected.

**Conclusion:**

Perioperative systemic glucocorticoids appear to modestly reduce early pain, though this reduction did not reach the MCID threshold. They were also associated with reduced PONV, shorter hospital stays, and lower inflammatory markers, without increasing short-term wound complications or infections in predominantly non-diabetic patients. Glycemic levels were not meaningfully affected in this population. Evidence for opioid-sparing effects remains inconsistent, and these results should be considered hypothesis-generating rather than definitive. Critically, these findings should not be extrapolated to diabetic patients, and routine use in this high-risk population cannot be recommended without targeted prospective trials. Future RCTs are needed to determine optimal dosing—single intravenous dexamethasone 10–20 mg represents a reasonable candidate for investigation—and to confirm safety in high-risk populations before formal clinical guidance can be established.

**Supplementary Information:**

The online version contains supplementary material available at 10.1186/s42836-026-00397-4.

## Introduction

Total hip arthroplasty (THA) is a well-established intervention for end-stage hip osteoarthritis, offering reliable pain relief and functional restoration. With aging populations and rising global prevalence of osteoarthritis, demand for THA continues to increase—projected to rise by over 70% in the United States alone by 2030 [1]. Despite advances in surgical technique and perioperative care, recovery after THA remains challenging. Persistent postoperative pain often delays mobilization and increases opioid dependence [2]. Postoperative nausea and vomiting (PONV) affect 30–50% of patients, impairing oral intake and patient satisfaction [3]. Additionally, perioperative glycemic fluctuations—particularly in diabetic or metabolically vulnerable patients—may elevate risks of wound complications and prolonged hospitalization [4]. Perioperative glucocorticoids—particularly dexamethasone—are increasingly incorporated into enhanced recovery protocols due to their multimodal effects: reducing inflammation, attenuating pain, and preventing PONV [5].

While clinical trials suggest benefits, findings are heterogeneous, likely reflecting variations in dosing, timing, and patient selection. This inconsistency is compounded by a critical limitation in the existing evidence synthesis: prior systematic reviews and meta-analyses have predominantly combined data from THA and total knee arthroplasty (TKA), despite important clinical differences between the two procedures [6]. Consequently, recommendations derived from combined analyses lack precision for the THA population. Furthermore, key safety concerns—especially regarding postoperative glycemic control and infection risk—remain inadequately addressed for patients undergoing THA.

Therefore, a definitive, procedure-specific synthesis of evidence is needed. We conducted this GRADE-assessed meta-analysis of RCTs with three primary objectives: (1) to provide a precise estimate of the efficacy of perioperative systemic glucocorticoids on THA-specific outcomes critical to ERAS, including pain (particularly during walking), PONV, and length of stay; (2) to establish a robust safety profile, with focused analysis on glycemic impact and surgical complications; and (3) to identify unresolved clinical questions—notably optimal dosing and safety in diabetic patients—to inform the design of future randomized trials.

## Methods

### Search strategy

This systematic review and meta-analysis were conducted in accordance with the Preferred Reporting Items for Systematic Reviews and Meta-Analyses (PRISMA) guidelines [7]. The completed PRISMA checklist is provided as Supplementary Material. The protocol was prospectively registered on PROSPERO (ID: CRD420251155587). PubMed, Web of Science, Scopus, and the Cochrane Library were searched from their inception to October 17, 2025, with no time restrictions applied to ensure comprehensive coverage of the relevant literature. The search strategy combined Medical Subject Headings (MeSH) and free-text keywords related to total hip arthroplasty and glucocorticoids. The full electronic search strings, including all Boolean operators, for each database are provided in Supplementary Table S1. No grey literature searches were initially performed, as the primary focus was on English-language peer-reviewed randomized controlled trials (RCTs) to ensure high internal validity and methodological consistency across the included studies. However, we recognize that this approach, along with our English-language restriction, could lead to publication and language bias. To address this limitation, the reference lists of eligible papers and relevant reviews were examined for additional records.

### Inclusion and exclusion criteria

Eligible studies were randomized controlled trials (RCTs), published as full-text articles in peer-reviewed journals. The population consisted of adult patients undergoing primary unilateral total hip arthroplasty under either general or regional anesthesia, regardless of indication. The intervention involved perioperative systemic glucocorticoids (e.g., dexamethasone, betamethasone, methylprednisolone, hydrocortisone, prednisolone, prednisone, triamcinolone), administered within 24 h preoperatively, intraoperatively, or on the day of surgery. Eligible studies were required to have a control group receiving either a placebo or standard care without systemic glucocorticoids. Studies were required to report at least one of the following primary outcomes: glycemic control, PONV, range of motion (ROM), functional recovery, opioid consumption, or acute pain outcomes. Secondary outcomes included inflammatory response, hospital resource use, and adverse events. Exclusion criteria included non-RCT designs, bilateral or revision arthroplasties, other joint procedures (e.g., knee), local or intra-articular steroids without systemic use, the absence of relevant outcome data, pediatric populations, and animal studies. Two authors independently screened records using Rayyan, resolving disagreements by discussion with a third author.

### Data extraction

Data were extracted independently by two authors using a predefined form. Extracted details covered study design, sample size, funding, patient demographics (age, sex, prevalence of diabetes), intervention (drug, dose, route, timing), comparator, and outcomes (means, SDs, odds ratios with 95% CIs). Continuous data were recorded as means and SDs where available; medians with interquartile ranges were converted to means and SDs using validated methods when necessary. Differences between authors were resolved by consensus.

### Quality assessment

The Cochrane Risk of Bias 2 (RoB 2) tool [8] was used to evaluate study quality across five domains: randomization process, deviations from intended interventions, missing outcome data, outcome measurement, and selection of the reported results. Two authors assessed independently, with disagreements resolved by consensus. Additional consideration was given to incomplete reporting of baseline characteristics or concomitant medications, which—while not directly captured in RoB 2 domains—may introduce residual confounding. However, randomization is expected to balance such unmeasured factors across groups. (Fig. 2) The certainty of evidence for selected critical outcomes (VAS pain at rest, VAS pain during walking, postoperative glucose levels, PONV incidence, and infection rates) was assessed using the Grading of Recommendations Assessment, Development and Evaluation (GRADE) framework, considering risk of bias, inconsistency, indirectness, imprecision, and publication bias [9]. The judgments were carefully reevaluated, with outcomes downgraded due to inconsistencies. The final assessments, along with detailed justifications, are presented in Tables 3 and 4.

### Statistical analysis

All statistical analyses were conducted in accordance with the Cochrane Handbook for Systematic Reviews of Interventions, with the primary aim of estimating the overall effect of systemic glucocorticoid administration on postoperative outcomes. For continuous variables (e.g., glucose levels, VAS pain scores, blood loss, hospital stay, and inflammatory markers), mean differences (MDs) were calculated when measurement scales were uniform. Dichotomous outcomes (e.g., incidence of PONV or infections) were summarized using odds ratios (ORs). For outcomes such as postoperative nausea and vomiting (PONV), both composite measures (e.g., “any PONV”) and individual components (e.g., nausea severity, rescue antiemetic use) were analyzed and reported separately to provide a comprehensive picture of the intervention’s effects. All effect sizes were reported with 95% confidence intervals (CIs). Between-study heterogeneity was assessed using Cochran’s Q test (*p* < 0.10 indicating significance) and quantified with the I^2^ statistic, interpreted as low (< 40%), moderate (40–75%), or high (> 75%). Given the expected clinical and methodological diversity, a random-effects model based on the DerSimonian-Laird method was used for all pooled analyses. Pre-specified subgroup analyses were undertaken according to the timing of outcome assessment (e.g., postoperative days 1–3), type of analgesic used, and outcome domain (e.g., range of motion in different directions), with subgroup differences formally tested using chi-squared statistics. Assessment of publication bias was not feasible because no outcome included ≥ 10 studies, consistent with Cochrane and Egger test recommendations that funnel plot–based methods are unreliable with fewer than ten studies. All analyses were performed using Stata version 17 (StataCorp, College Station, TX, USA), and statistical significance was set at a two-sided *p* < 0.05. To explore and address sources of substantial heterogeneity (I^2^ > 75%), pre-specified sensitivity analyses were conducted, including leave-one-out analyses and the removal of potential outlier studies identified through visual inspection of forest plots. Furthermore, we adopted a cautious interpretive approach. Instead of using alternative random-effects estimators such as Hartung–Knapp or REML, which, although more conservative in confidence interval estimation, do not address the underlying clinical or methodological heterogeneity causing the high I^2^ values, we emphasize that pooled effect estimates for these outcomes should be seen as indicating a general direction of benefit rather than as precise effect sizes suitable for clinical decision-making.

## Results

### Search and screening

**Fig. 1:**
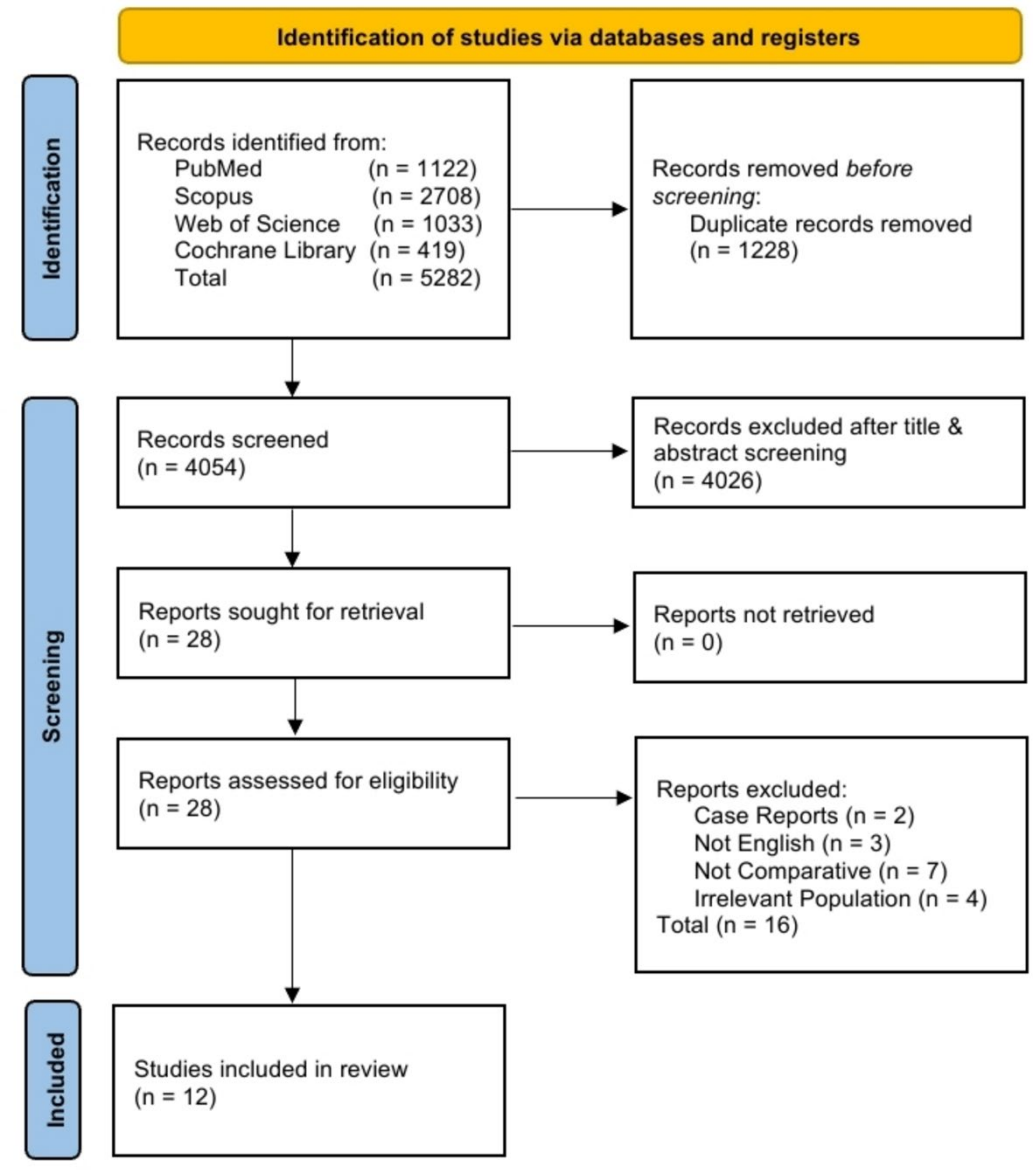
PRISMA flowchart of the selection process

The search retrieved 1,122 records from PubMed, 1,033 from Web of Science, 2,708 from Scopus, and 419 from Cochrane Library, totaling 5,282 records across all databases. After removing 1,228 duplicates, 4,054 records underwent title and abstract screening, during which 4,026 were excluded. A full-text review of 28 studies resulted in the inclusion of 12 trials [10–21]. The study selection process is detailed in the PRISMA 2020 flow diagram. (Fig. 1).

### Study characteristics

We analyzed 12 studies [10–21] on this topic, all of which were randomized controlled trials published between 2007 and 2024, encompassing a total of 1,128 patients. These studies were conducted across multiple countries, including China, Denmark, Canada, Australia, Poland, Iran, and the United States. The majority of studies were well-designed, employing double-blind, placebo-controlled methodologies to minimize bias. Participants were adults undergoing primary unilateral total hip arthroplasty. Mean patient age typically ranged between 60 and 72 years, although one study by Shafiei et al. [18] included a notably younger cohort with a mean age of approximately 44 years. The gender distribution across studies was generally balanced. Most participants were in relatively good preoperative health, commonly classified as American Society of Anesthesiologists (ASA) physical status I, II, or III. A key methodological feature was the application of strict exclusion criteria to ensure homogeneity among study populations. For instance, seven studies excluded patients with diabetes mellitus. However, two studies [11, 21] did include diabetic patients, providing valuable data for subgroup analysis. Other standard exclusion criteria included rheumatoid arthritis, active infection, significant cardiac, renal, or hepatic disease, and prior glucocorticoid use within 3 to 6 months before surgery. Surgical and anesthetic techniques varied across studies but adhered to standard institutional protocols. Anesthesia was administered as general, spinal, or epidural, often determined by hospital practice or anesthesiologist preference. Follow-up duration also varied considerably depending on the outcomes assessed. Some studies monitored patients for only 24 to 72 h postoperatively, while others followed them for 3 to 6 months to evaluate functional recovery and complication rates. One study extended follow-up to 12 months to assess longer-term outcomes. A detailed summary of study-level characteristics is provided in Table 1.

**Table 1:** Summary and baseline characteristics of included studies

Study ID	Country	Study Design	Sample Size	Follow-up	Key Comorbidities	Age (Mean ± SD) (Glucocorticoids/Control)	BMI (Mean ± SD) (Glucocorticoids/Control)
Bergeron et al. 2009 [12]	Canada	RCT, double-blind, placebo-controlled	50	12 months	DM was excluded	NR	NR
Dissanayake et al. 2018 [20]	Australia	Double-blinded placebo-controlled RCT	164	6 weeks	DM was excluded	68.7 ± 9.9/66.7 ± 10.8	NR
Gądek et al. 2020	Poland	Double-blind RCT	77	3 days	HTN: 100% both; AF, IHD, COPD, CHF reported	72.66 ± 6.92/72.71 ± 5.93	NR
Kardash et al. 2008 [10]	Canada	RCT	50	1 month	DM was excluded	69.0 ± 7.2/68.8 ± 11.4	NR
Lindberg-Larsen et al. 2018 [15]	Denmark	Randomized, Double-Blind, Placebo-Controlled	59	24 h	Insulin-dependent DM was excluded	67.4 ± 5.4/67.2 ± 6.7	26.9 ± 4.1/27.5 ± 4.3
Lunn et al. 2012	Denmark	Double-blind, placebo-controlled RCT	48	1 month	Diabetic neuropathy was excluded	66.33 ± 21.28/64.67 ± 22.07	27 ± 4/27 ± 5
Mo et al. 2024 [21]	China	RCT	98	3 months	HTN: 11/50 vs 10/48; DM: 3/50 vs 2/48	64.36 ± 5.64/64.19 ± 5.84	24.73 ± 3.05/24.82 ± 3.20
Shafiei et al. 2022 [18]	Iran	Double-Blind, RCT	70	6 months	Poorly controlled DM and HTN were excluded	44.3 ± 15.2/44.6 ± 16.7	NR
Sculco et al. 2015	USA	Prospective Double-Blind Placebo-Controlled Trial	27	3 months	DM was excluded	66.21 ± 8.80/65.13 ± 7.16	28.03 ± 4.86/26.04 ± 3.92
Lei et al. 2017-1	China	RCT	110	2 weeks	HTN: 18 vs 18	53.40 ± 13.44/56.64 ± 13.02	23.28 ± 3.09/24.29 ± 3.51
Lei et al. 2020 [11]	China	Randomized, blinded, placebo-controlled trial	165	3 months	HTN: 31/110 vs 16/55; DM: 13/110 vs 6/55	57.71 ± 10.14/58.76 ± 12.20	23.29 ± 2.87/24.26 ± 3.46
Lei et al. 2017-2	China	Prospective RCT	210	3 months	Not specified	56.10 ± 10.08/57.50 ± 12.00	23.15 ± 2.90/23.80 ± 3.40

### Interventional characteristics

The primary focus of the included studies was the administration of glucocorticoids in the perioperative period. all other aspects of care—including analgesic and antiemetic regimens—were standardized within each trial to isolate the effects of glucocorticoids. There was considerable variation in the type, dose, and route of glucocorticoid administration across studies. dexamethasone was the most commonly used agent, appearing in 7 studies with doses ranging from 8 to 40 mg. four studies employed methylprednisolone, typically at higher doses (40 mg or 125 mg). one study [16] by Sculco et al. (2015) utilized a distinct regimen: Oral prednisone (20 mg) was administered preoperatively, followed by two intravenous doses of hydrocortisone (100 mg each) postoperatively. Glucocorticoids were predominantly administered intravenously. In some trials, a single dose was given at the time of anesthesia induction; in others, an initial dose was followed by a second dose 24 h later. Comparator groups were carefully designed. In all the included studies, the control group received a placebo (isotonic saline) administered via the same route as the active intervention. To ensure consistency in postoperative management, all studies adhered to standardized protocols for controlling pain and nausea. patients routinely received scheduled non-opioid analgesics—such as acetaminophen and NSAIDs (e.g., celecoxib or diclofenac)—and occasionally gabapentinoids. more potent opioids—including morphine (via patient-controlled analgesia) or oxycodone (oral)—were reserved for breakthrough pain exceeding a predefined threshold (e.g., ≥ 4 on a 10-point pain scale). Similarly, antiemetics (e.g., metoclopramide or ondansetron) were administered only on an as-needed basis for symptomatic nausea or vomiting. Full details of interventions and controls for each study are provided in Table 2.

**Table 2:** Interventional characteristics of the included studies

Study ID	Route of Administration	Glucocorticoid Type	Glucocorticoid Dose & Timing/Control	Anesthesia Type (Glucocorticoids/Control)
Bergeron et al. 2009 [12]	IV	Dexamethasone	40 mg IV pre-op [after paresthesia]/placebo	Spinal/Spinal
Dissanayake et al. 2018 [20]	IV	Dexamethasone	8 mg IV at induction + 8 mg IV at 24 h/saline	Spinal & general/Spinal & general
Gądek et al. 2020	IV	Methylprednisolone	125 mg IV preemptive/saline	Spinal/Spinal
Kardash et al. 2008 [10]	IV	Dexamethasone	40 mg IV pre-op/placebo	Spinal/Spinal
Lindberg-Larsen et al. 2018 [15]	IV	Methylprednisolone	125 mg IV post-spinal/saline	Spinal/Spinal
Lunn et al. 2012	IV	Methylprednisolone	125 mg IV pre-op/saline	Spinal/Spinal
Mo et al. 2024 [21]	IV	Dexamethasone	10 mg IV pre-op/saline	Intraspinal/Intraspinal
Shafiei et al. 2022 [18]	IV	Methylprednisolone acetate	125 mg IV after induction/saline	General/General
Sculco et al. 2015	Oral + IV	Prednisone + Hydrocortisone	Prednisone 20 mg PO [equiv. 100 mg hydrocortisone] 2 h pre-op + 2 × 100 mg IV 8 h/placebo	Spinal & Epidural/Spinal & Epidural
Lei et al. 2017-1	IV	Dexamethasone	10 mg IV × 2 [post-induction + post-op]/placebo	General/General
Lei et al. 2020 [11]	IV	Dexamethasone	20 mg IV single dose and 10 mg IV × 2 [post-induction + post-op]/placebo	General/General
Lei et al. 2017-2	IV	Dexamethasone	10 mg IV multiple doses/placebo	General/General

### Quality assessment & evidence certainty

**Fig. 2:**
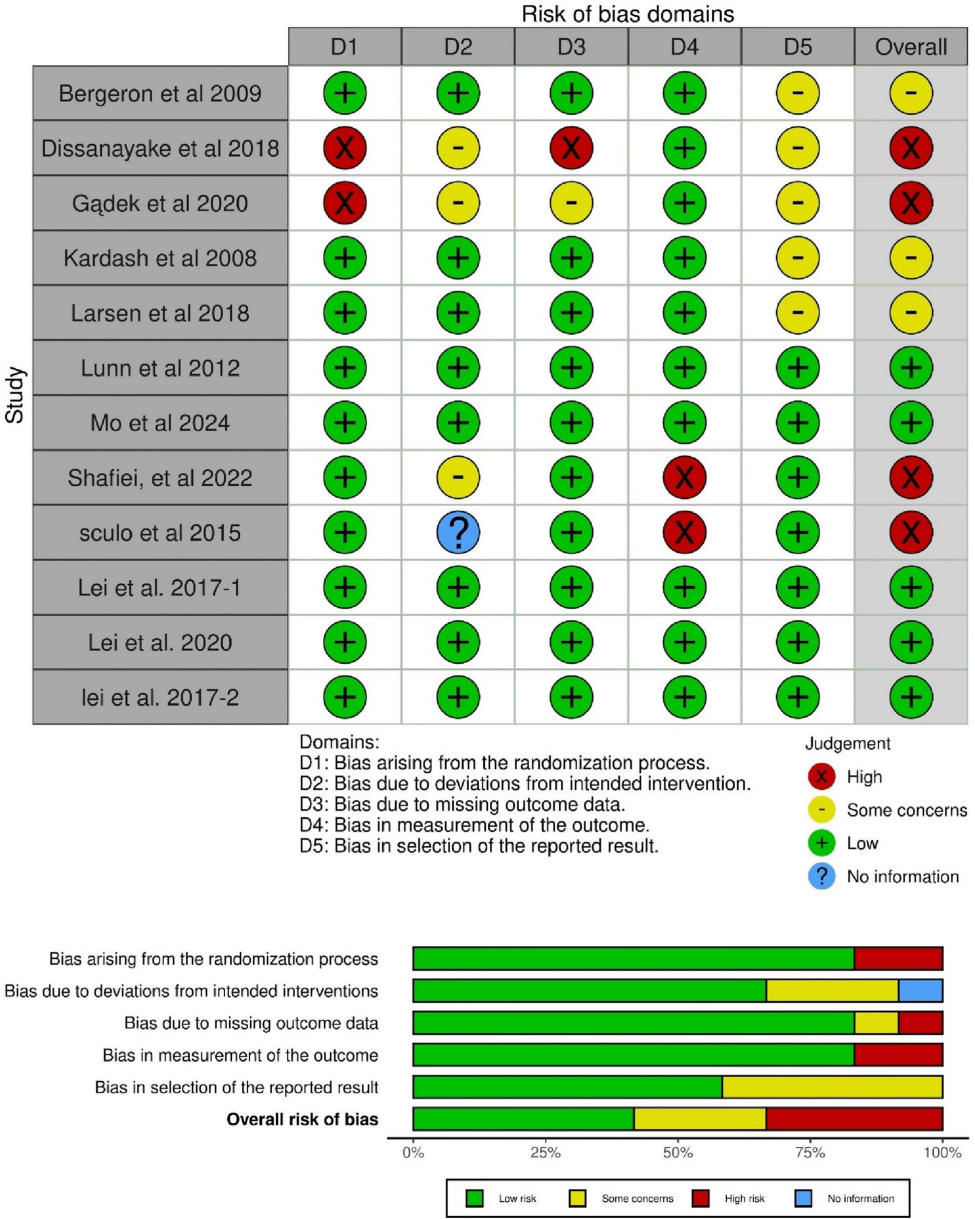
Risk of bias assessment of the included studies

The overall risk of bias across the included trials was evaluated using the Cochrane RoB 2 tool. Of the included RCTs, five were judged to have a low overall risk of bias, three raised “some concerns,” and four were rated at a high risk of bias. Concerns most frequently arose from the domains related to missing outcome data (D3) and selective reporting of results (D5). Specifically, the studies by Dissanayake et al. [20] and Gądek et al. [14] were rated as having “some concerns” due to incomplete outcome data for secondary endpoints. The study by Shafiei et al. [18] was judged to be at high risk of bias due to significant deviations from the intended intervention protocol that were not appropriately addressed in the analysis. A detailed summary of the domain-level RoB 2 assessment for each included study is presented in Fig. 2. The certainty of evidence for critical outcomes was subsequently assessed using the GRADE framework, incorporating these risk-of-bias judgments alongside assessments of inconsistency, indirectness, imprecision, and publication bias. The resulting evidence profiles are presented in Table 3, with a summary of findings in Table 4. In brief, moderate certainty evidence may support the efficacy of glucocorticoids for reducing pain during walking and at rest. Evidence for the incidence of PONV was also moderate. However, the certainty of evidence for infection rates was downgraded to low due to imprecision. Similarly, the evidence for the effect on postoperative glucose levels was also assessed as low certainty, reflecting both risk of bias concerns in contributing studies and imprecision. These GRADE assessments directly inform the strength of the conclusions drawn in this meta-analysis.

**Table 3:** GRADE evidence profile for the included randomized controlled trials

Certainty assessment							No of patients		Effect		Certainty	Importance
**No of studies**	**Study design**	**Risk of bias**	**Inconsistency**	**Indirectness**	**Imprecision**	**Other considerations**	**Glucocorticoids**	**Non-glucocorticoids**	**Relative** **(95% CI)**	**Absolute** **(95% CI)**		
**VAS at rest**												
6	randomised trials	serious	not serious	not serious	not serious	very strong association	465	330	MD − 0.20 (− 0.38 to − 0.01)	MD 0.20 lower (0.38 lower to 0.01 lower)	⨁⨁⨁◯ Moderate	CRITICAL
**VAS during walk**												
4	randomised trials	not serious	not serious	not serious	not serious	very strong association	355	228	MD − 0.50 (− 0.89 to − 0.12)	MD 0.50 lower (0.89 lower to 0.12 lower)	⨁⨁⨁◯ Moderate	CRITICAL
**Postoperative glucose level**												
5	randomised trials	serious^a^	not serious	not serious	serious^b^	none	283	220	MD − 0.01 (− 0.15 to 0.13)	MD 0.01 mmol/L lower (0.15 lower to 0.13 higher)	⨁⨁◯◯ Low^a,b^	CRITICAL
**PONV incidence**												
4	randomised trials	not serious	not serious	not serious	not serious	very strong association	16/355 (4.5%)	53/228 (23.2%)	OR 0.21 (0.13 to 0.35)	217 fewer per 1,000 (from 247 to 170 fewer)	⨁⨁⨁◯ Moderate	CRITICAL
**Infection rate**												
7	randomised trials	serious	not serious	not serious	serious^b^	none	1/500 (0.2%)	2/366 (0.5%)	OR 0.66 (0.17 to 2.53)	80 fewer per 1,000 (from 232 fewer to 220 more)	⨁⨁◯◯ Low^a,b^	CRITICAL

CI: confidence interval; MD: mean difference; OR: odds ratio

^a^Most of the studies are at high risk of bias

^b^Wide 95% CI

**Table 4:** Summary of findings for the effect of perioperative glucocorticoids on postoperative outcomes

Outcomes	Anticipated absolute effects* (95% CI)		Relative effect (95% CI)	No of participants (studies)	Certainty of the evidence (GRADE)	Comments
	**Risk with [comparison]**	**Risk with [intervention]**				
VAS at rest	The mean VAS at rest was 0	MD 0.20 lower (0.38 lower to 0.01 lower)	MD − 0.20 (− 0.38 to − 0.01)	795 (6 RCTs)	⨁⨁⨁◯ Moderate	Glucocorticoids may result in modest differences in VAS at rest
VAS during walk	The mean VAS during walk was 0	MD 0.50 lower (0.89 lower to 0.12 lower)	MD − 0.50 (− 0.89 to − 0.12)	583 (4 RCTs)	⨁⨁⨁◯ Moderate	Glucocorticoids may result in a large reduction in VAS during walking
Postoperative Glucose Level	The mean Postoperative Glucose Level was 0 mmol/L	MD 0.01 mmol/L lower (0.15 lower to 0.13 higher)	MD − 0.01 (− 0.15 to 0.13)	503 (5 RCTs)	⨁⨁◯◯ Low^a,b^	Glucocorticoids may result in little to no difference in Postoperative Glucose Level
PONV incidence	232 per 1,000	60 per 1,000 (38 to 96)	OR 0.21 (0.13 to 0.35)	583 (4 RCTs)	⨁⨁⨁◯ Moderate	Glucocorticoids may result in a large reduction in PONV incidence
Infection Rate	232 per 1,000	166 per 1,000 (49 to 433)	OR 0.66 (0.17 to 2.53)	866 (7 RCTs)	⨁⨁◯◯ Low^a,b^	Glucocorticoids may result in little to no difference in Infection Rate

^*^The risk in the intervention group (and its 95% confidence interval) is based on the assumed risk in the comparison group and the relative effect of the intervention (and its 95% CI)

^a^Most of the studies are at high risk of bias

^b^Wide 95% CI

CI: confidence interval; MD: mean difference; OR: odds ratio

GRADE working group grades of evidence:

High certainty: We are very confident that the true effect lies close to that of the estimate of the effect

Moderate certainty: We are moderately confident in the effect estimate: the true effect is likely to be close to the estimate of the effect, but there is a possibility that it is substantially different

Low certainty: our confidence in the effect estimate is limited; the true effect may be substantially different from the estimate of the effect

Very low certainty: we have very little confidence in the effect estimate; the true effect is likely to be substantially different from the estimated effect

### Primary outcomes

**Fig. 3:**
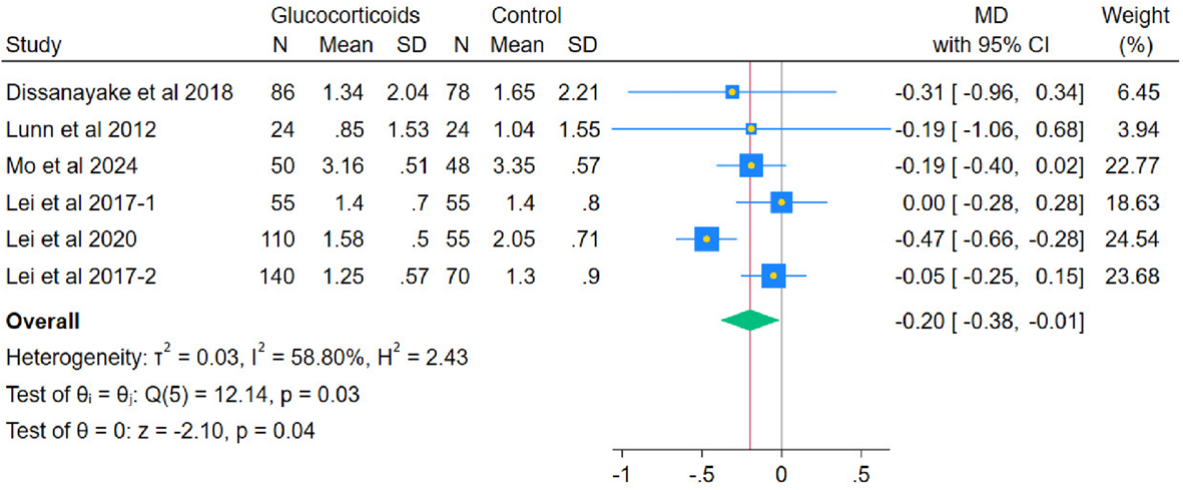
Forest plot illustrating the mean difference in postoperative VAS at rest at the last reported time point

**Fig. 4:**
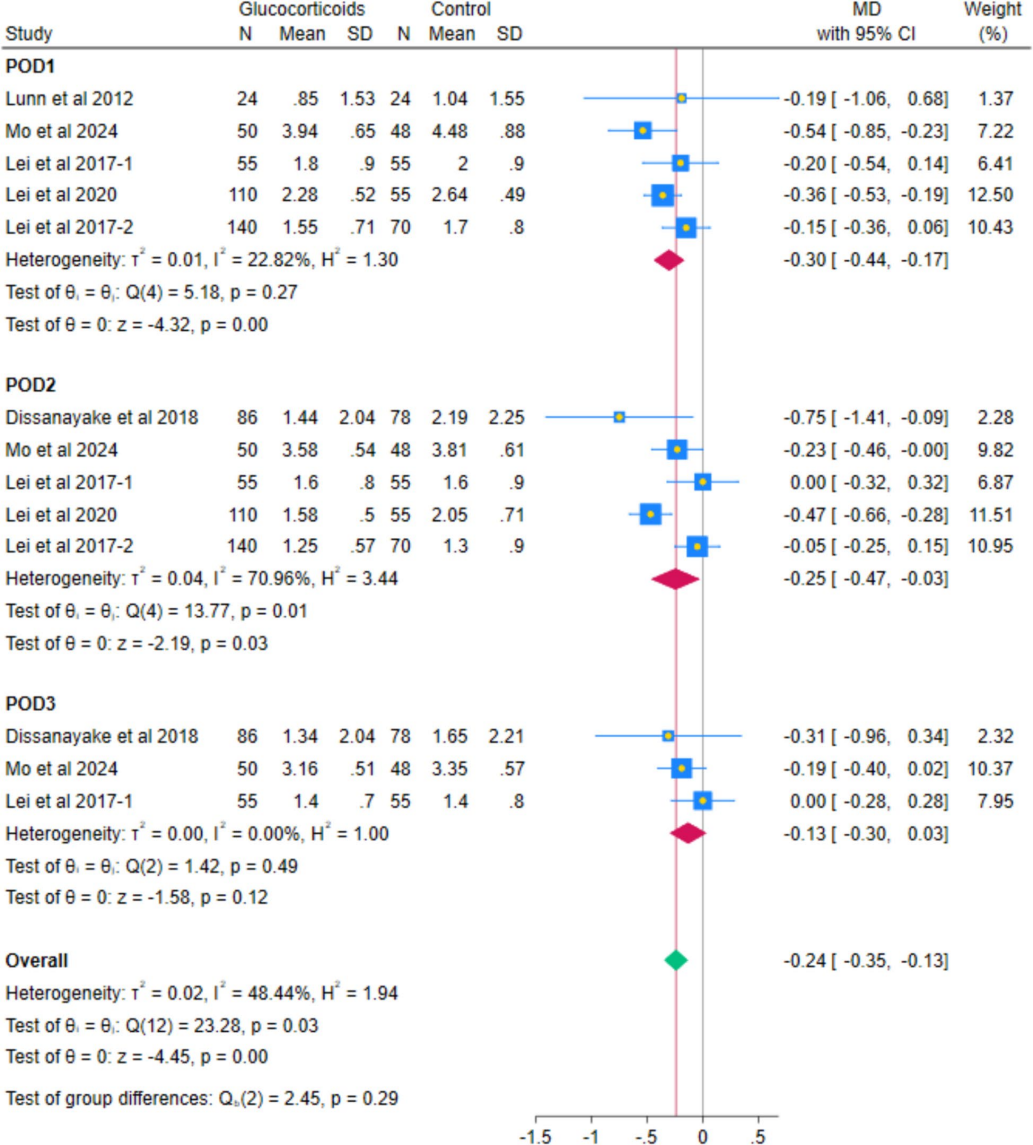
Forest plot illustrating the subgroup analysis of postoperative VAS at rest at different follow-up intervals

**Fig. 5:**
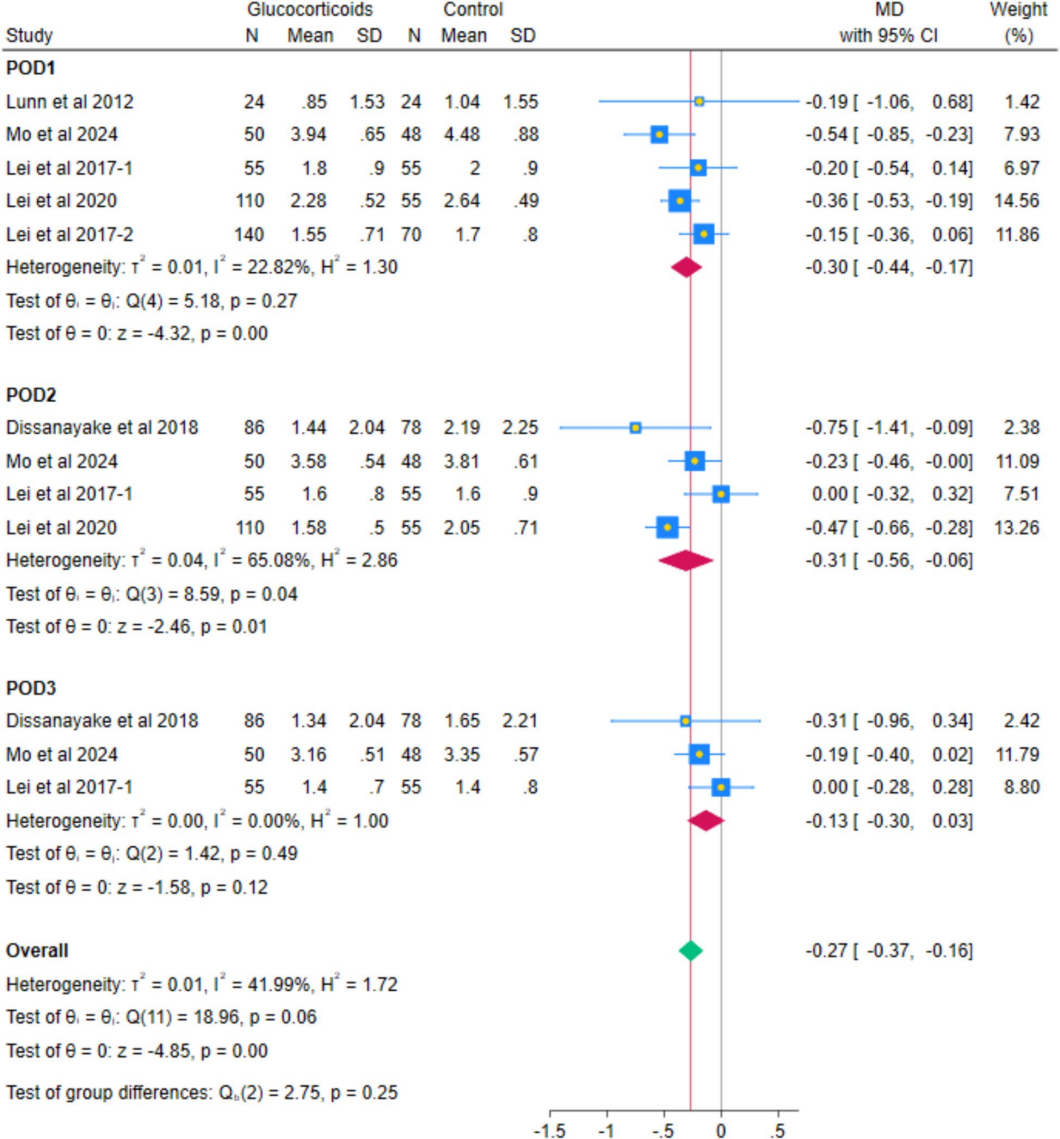
Forest plot illustrating the subgroup analysis of postoperative VAS at rest after excluding Lei et al. 2017-2 from the 2nd postoperative day

The pooled analysis of 6 RCTs showed a statistically significant reduction in VAS pain at rest with systemic glucocorticoids compared with controls (MD − 0.20; 95% CI − 0.38 to − 0.01; *p* = 0.04; I^2^ = 58.80%). (Fig. 3) Subgroup analyses by postoperative day indicated reductions on day 1 (MD − 0.30, 95% CI − 0.44 to − 0.17; *p* < 0.001; I^2^ = 22.82%), day 2 (MD − 0.25, 95% CI − 0.47 to − 0.03; *p* = 0.03; I^2^ = 70.96%), but not for day 3 (MD − 0.13, 95% CI − 0.30 to 0.03; *p* = 0.12; I^2^ = 0.00%). (Fig. 4) All observed reductions fell below the Minimal Clinically Important Difference (MCID) threshold of − 1.86 cm on a VAS scale, indicating no clinically perceptible pain relief at rest. After excluding Lei et al. (2017-2), the heterogeneity decreased while maintaining the statistically significant difference at POD2 (MD − 0.31, 95% CI − 0.56 to − 0.06; *p* = 0.01; I^2^ = 65.08% (Fig. 5).

**Fig. 6:**
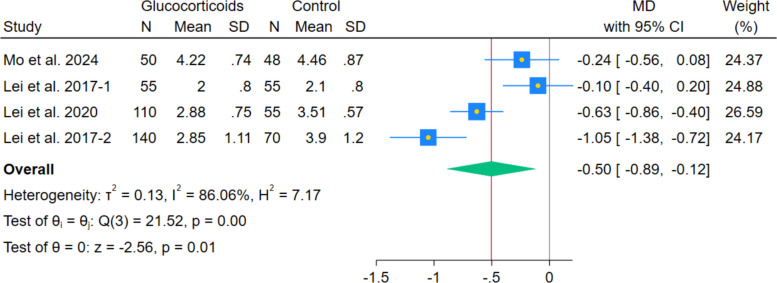
Forest plot illustrating the mean difference in postoperative VAS during walking at the last reported time point

**Fig. 7:**
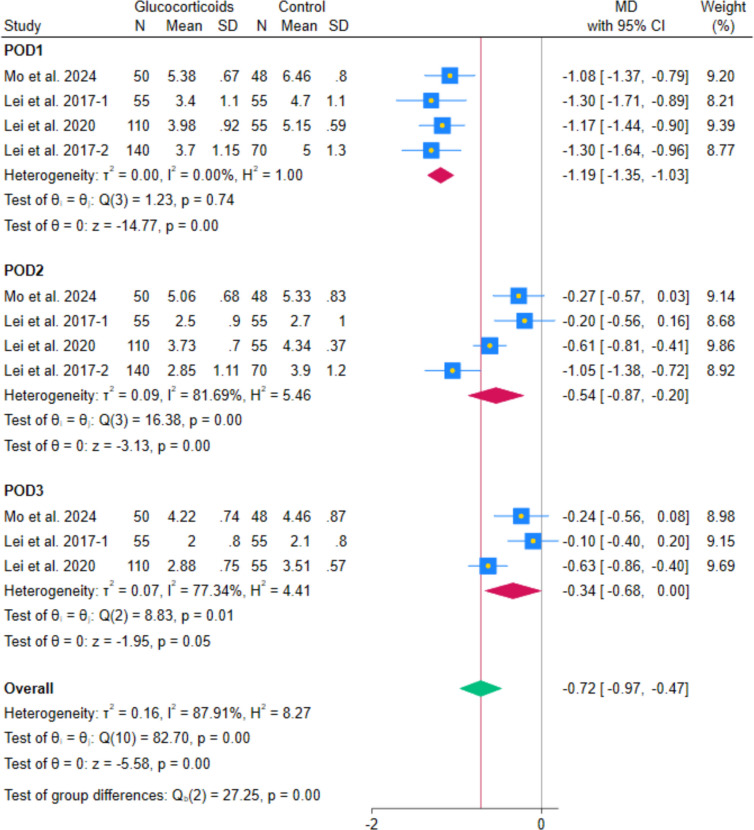
Forest plot illustrating the subgroup analysis of postoperative VAS during walking at different follow-up intervals

**Fig. 8:**
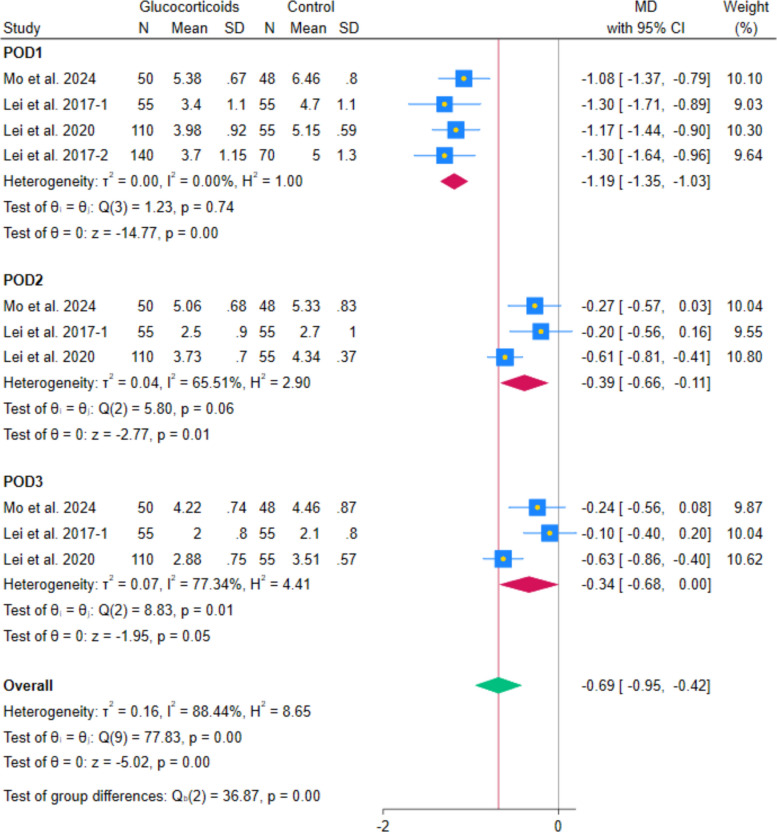
Forest plot illustrating the subgroup analysis of postoperative VAS during walking after excluding Lei et al. 2017-2 from the 2nd postoperative day

The pooled analysis of 4 RCTs showed a reduction in VAS pain during walking with systemic glucocorticoids compared with controls (MD − 0.50, 95% CI − 0.89 to − 0.12; *p* = 0.01; I^2^ = 86.06%). (Fig. 6) Subgroup analyses by postoperative day indicated reductions on day 1 (MD − 1.19, 95% CI − 1.35 to − 1.03; *p* < 0.001; I^2^ = 0.00%), day 2 (MD − 0.54, 95% CI − 0.87 to − 0.20; *p* < 0.001; I^2^ = 81.69%), but not for day 3 (MD − 0.34, 95% CI − 0.68 to 0.00; *p* = 0.05; I^2^ = 77.34%). (Fig. 7) After excluding Lei et al. 2017-2, the heterogeneity decreased while maintaining the statistically significant difference at POD2 (MD − 0.39, 95% CI − 0.66 to − 0.11; *p* = 0.01; I^2^ = 65.51%). (Fig. 8) The POD1 walking pain reduction (− 1.19 cm) fell below the established THA-specific MCID threshold of − 1.86 cm, indicating that even this most pronounced effect did not reach clinically meaningful levels. Leave-one-out sensitivity analyses maintained statistical significance in all cases.

**Fig. 9:**
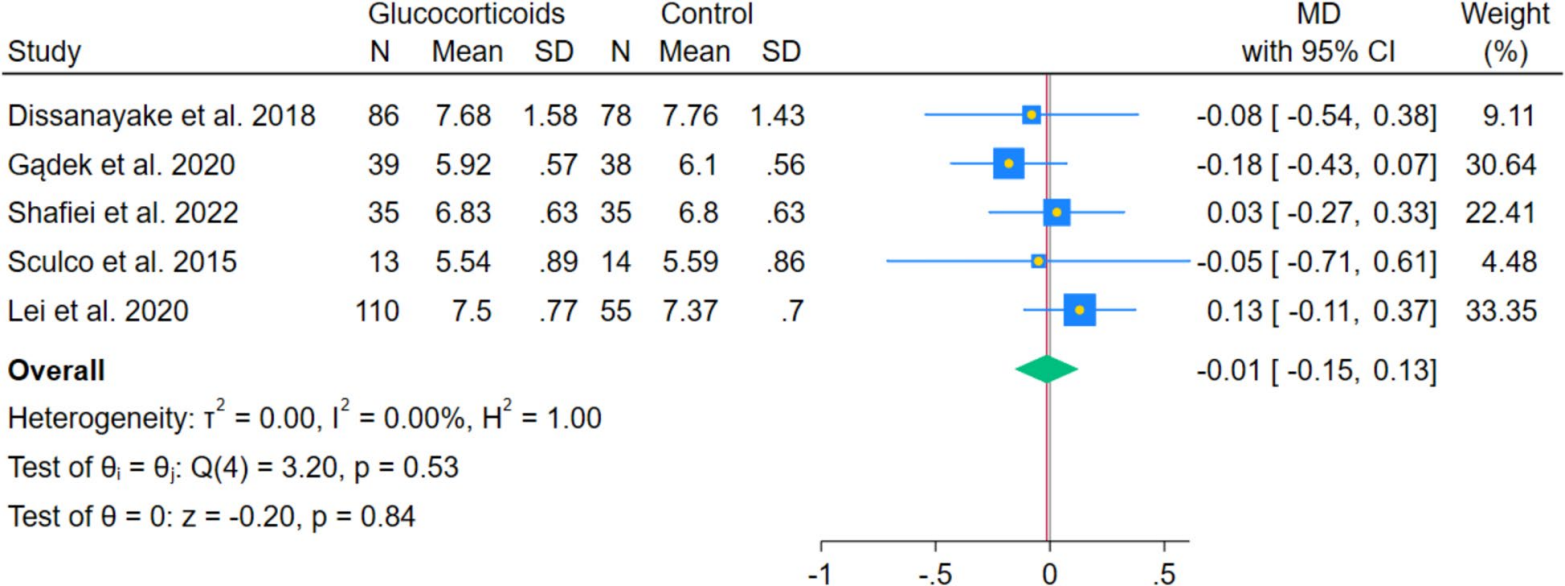
Forest plot showing the difference in postoperative glucose levels at the last reported time point

**Fig. 10:**
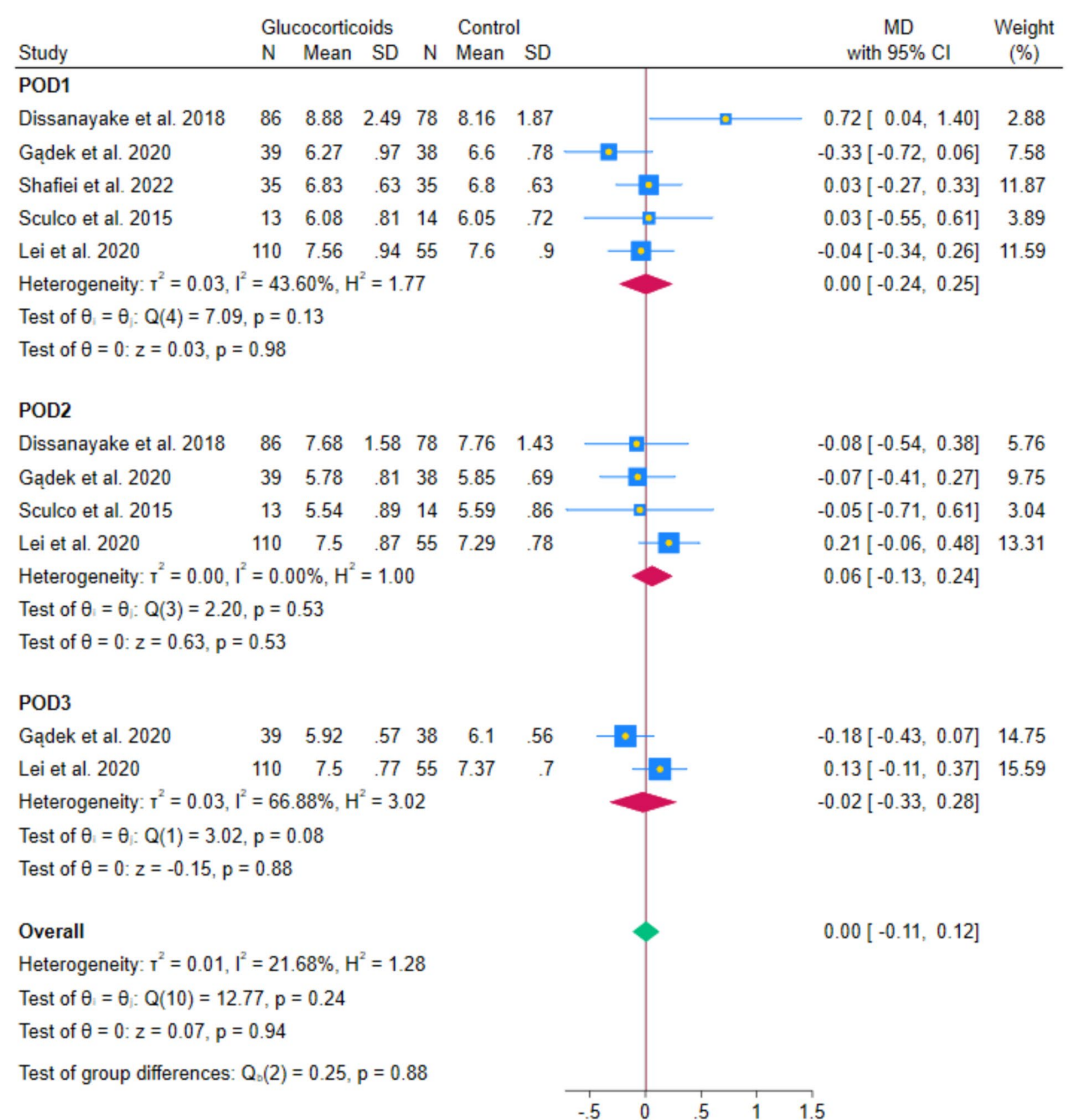
Forest plot showing the subgroup analysis of postoperative glucose levels at different follow-up intervals

The meta-analysis of 5 RCTs showed no statistically significant difference in postoperative glucose levels between systemic glucocorticoids and controls (MD − 0.01 mmol/L; 95% CI − 0.15 to 0.13; *p* = 0.84; I^2^ = 0.00%). (Fig. 9) Subgroup analyses by postoperative day indicated no statistically significant differences on day 1 (MD 0.00 mmol/L; 95% CI − 0.24 to 0.25; *p* = 0.98; I^2^ = 43.6%), day 2 (MD 0.06 mmol/L; 95% CI − 0.13 to 0.24; *p* = 0.53; I^2^ = 0.00%), and day 3 (MD − 0.02 mmol/L; 95% CI − 0.33 to 0.28; *p* = 0.88; I^2^ = 66.88%). (Fig. 10) Leave-one-out sensitivity analyses remained statistically non-significant in all cases.

**Fig. 11:**
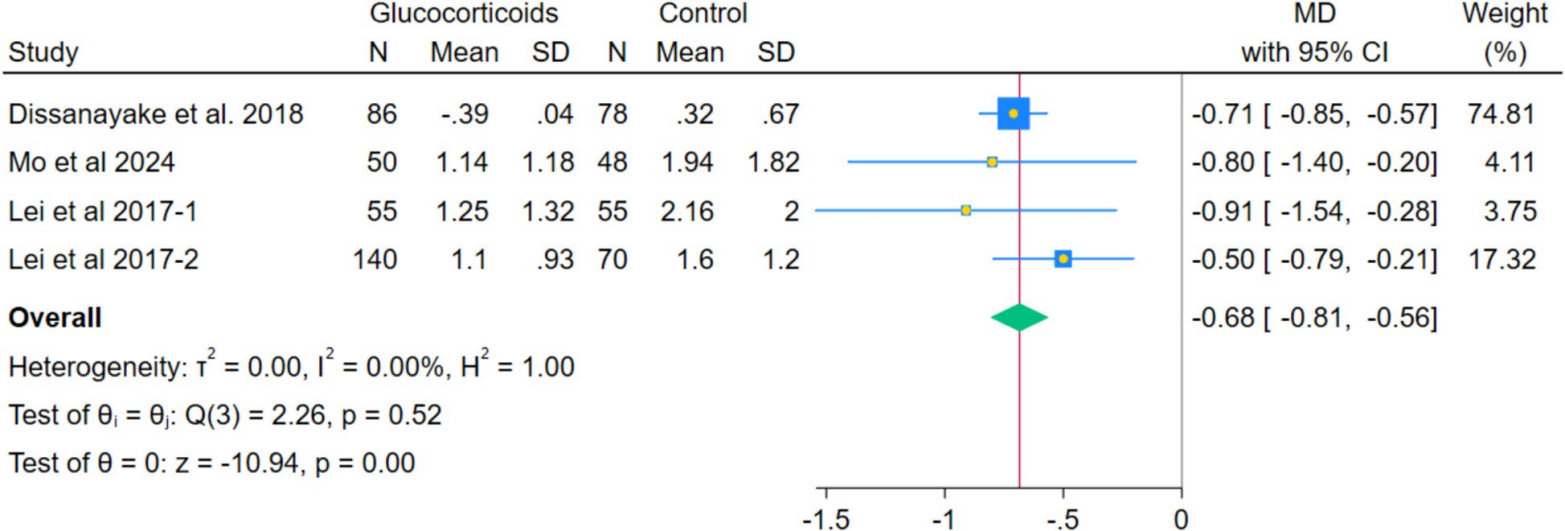
Forest plot illustrating the mean difference in postoperative nausea VAS score at the last reported time point

**Fig. 12:**
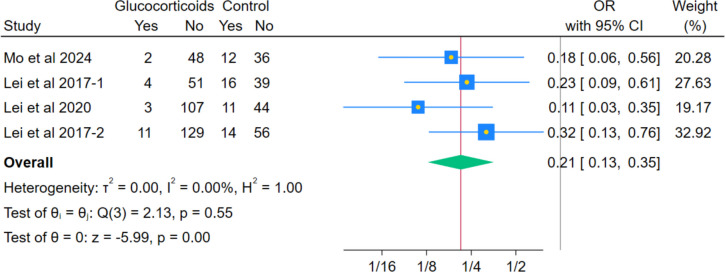
Forest plot illustrating the incidence of postoperative nausea and vomiting (PONV)

In the pooled data from 4 RCTs, systemic glucocorticoids showed a statistically significant decrease in nausea severity, as measured by VAS (MD − 0.68, 95% CI − 0.81 to − 0.56; *p* < 0.001; I^2^ = 0.00%). (Fig. 11) The meta-analysis indicated a statistically significant reduction in the odds of the composite outcome of overall postoperative nausea and vomiting (PONV) incidence among patients receiving systemic glucocorticoids versus controls (OR 0.21; 95% CI 0.13–0.35; *p* < 0.001; I^2^ = 0.00%), (Fig. 12). This composite effect is supported by significant reductions in key components, including nausea severity and the need for rescue antiemetics, as reported below. Results held in leave-one-out sensitivity analyses, remaining statistically significant across all exclusions.

**Fig. 13:**
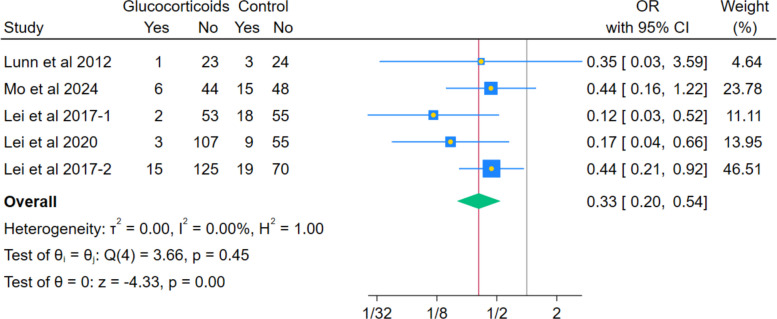
Forest plot illustrating the difference in rescue antiemetic use

The meta-analysis revealed a statistically significant decrease in the odds of rescue antiemetic use in the glucocorticoids group compared to the non-glucocorticoids group (OR 0.33, 95% CI 0.20–0.54; *p* < 0.001; I^2^ = 0.00%). (Fig. 13) The sensitivity analysis using the leave-one-out test remained statistically significant for all scenarios after removing each study.

**Fig. 14:**
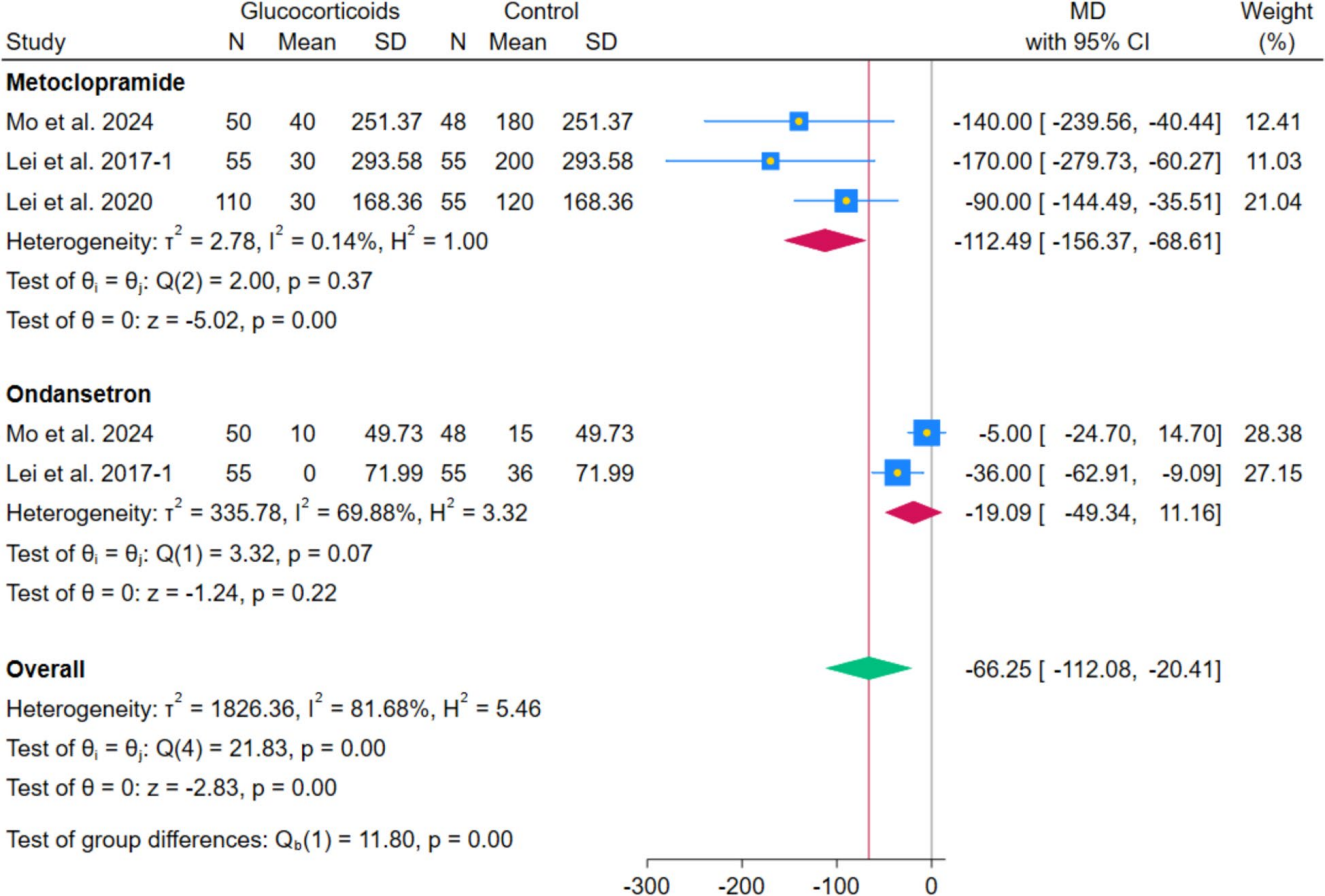
Forest plot illustrating the difference in postoperative metoclopramide and ondansetron consumption

The pooled analysis of 3 RCTs showed a statistically significant difference in metoclopramide consumption between systemic glucocorticoids and controls (MD − 112.49 mg, 95% CI − 156.37 to − 68.61; *p* < 0.001; I^2^ = 0.14%). In contrast, the pooled analysis of 2 RCTs showed no statistically significant difference in ondansetron consumption between systemic glucocorticoids and controls (MD − 19.09 mg, 95% CI − 49.34 to 11.16; *p* = 0.22; I^2^ = 69.88%). (Fig. 14).

**Fig. 15:**
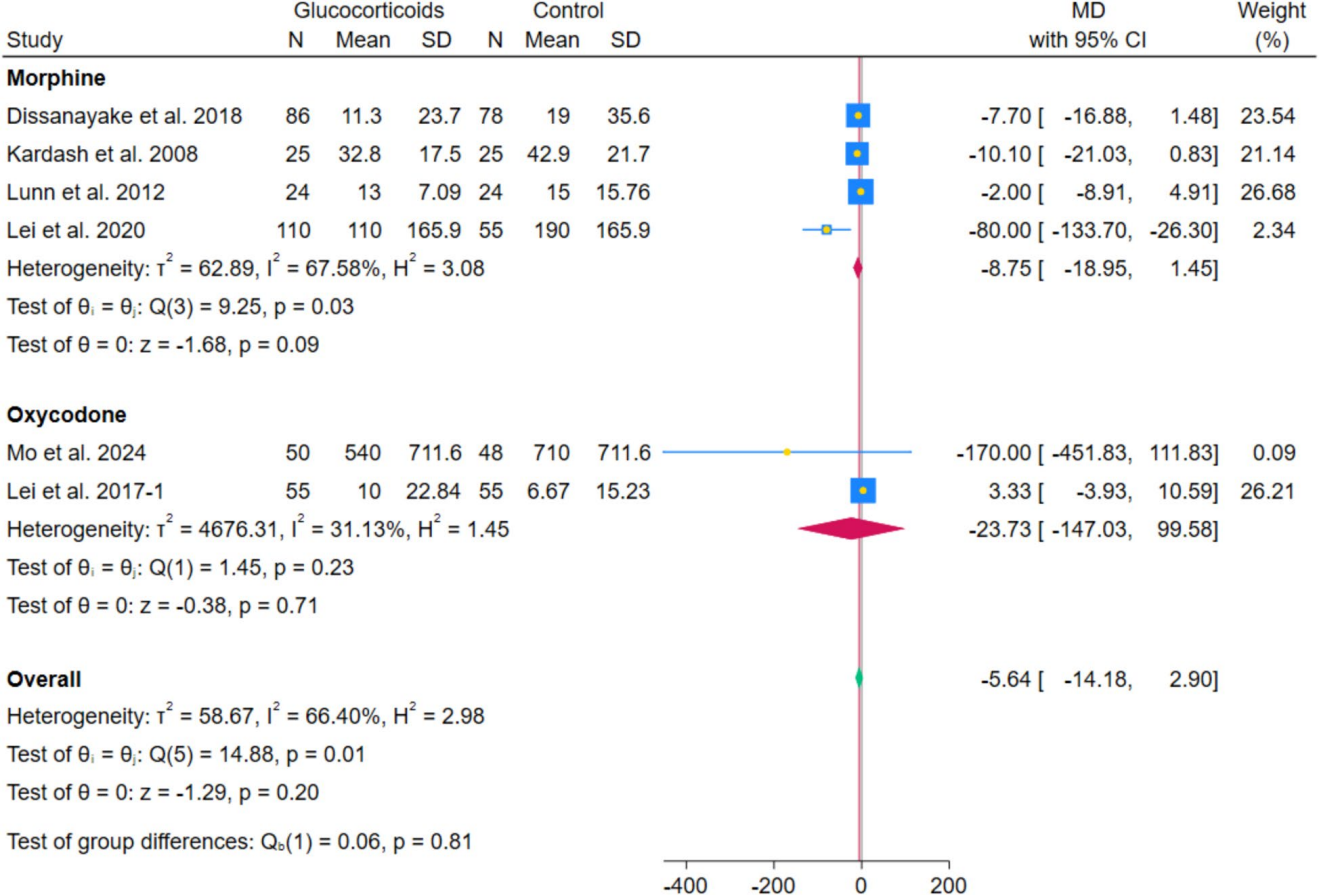
Forest plot illustrating the difference in morphine and oxycodone consumption

**Fig. 16:**
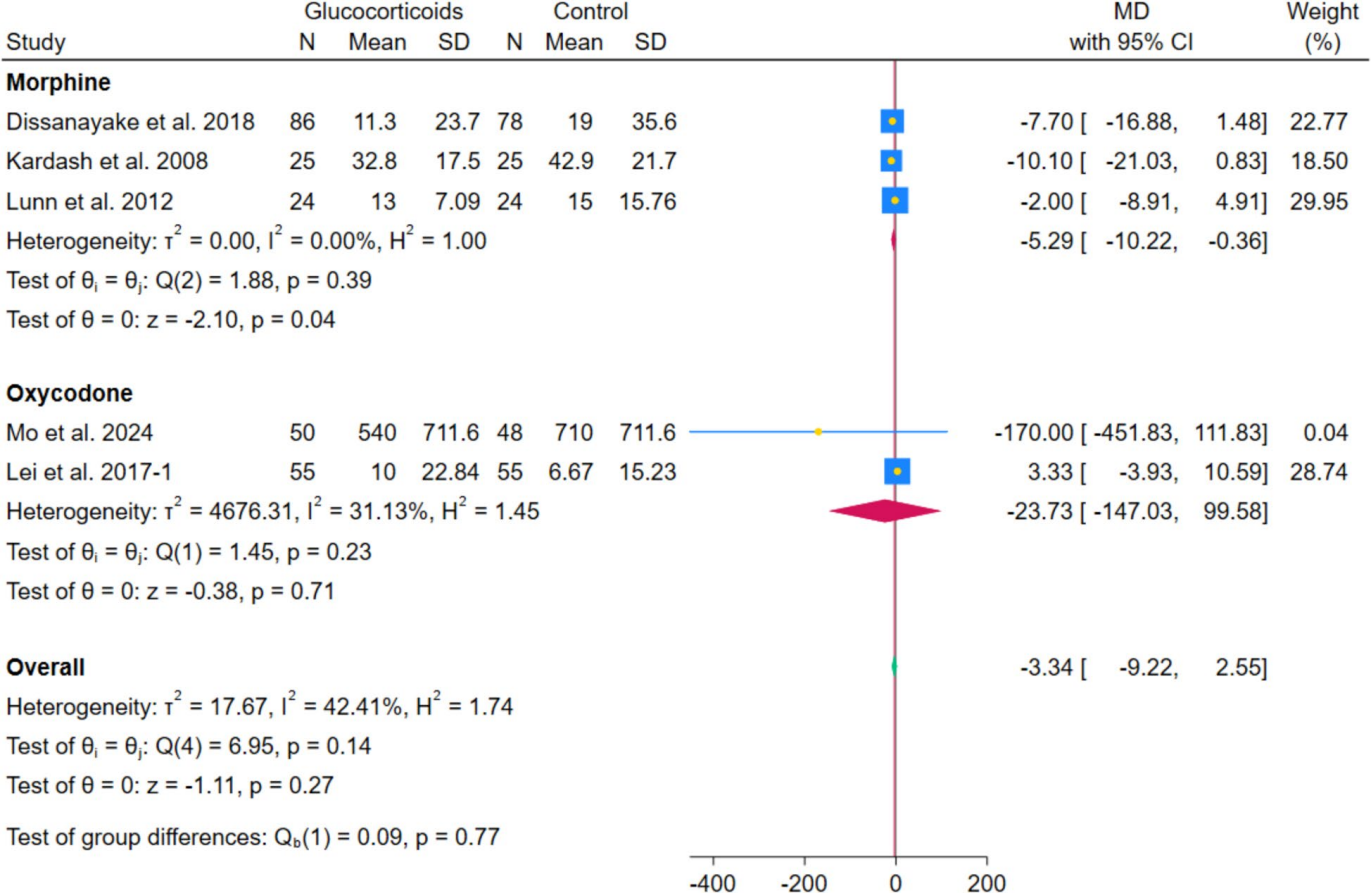
Forest plot illustrating the difference in morphine and oxycodone consumption after excluding Lei et al. 2020

The pooled analysis of 4 RCTs showed no statistically significant reduction in morphine consumption between systemic glucocorticoids and controls (MD − 8.75 mg; 95% CI − 18.95 to 1.45; *p* = 0.09; I^2^ = 67.58%). Similarly, the pooled analysis of 2 RCTs showed no statistically significant difference in oxycodone consumption between systemic glucocorticoids and controls (MD − 23.73 mg; 95% CI − 147.03 to 99.58; *p* = 0.71; I^2^ = 31.13%). (Fig. 15) However, after excluding Lei et al. 2020, the pooled analysis showed a statistically significant reduction in morphine consumption with no heterogeneity (MD − 5.29 mg; 95% CI − 10.22 to − 0.36; *p* = 0.04; I^2^ = 0.00%). (Fig. 16).

**Fig. 17:**
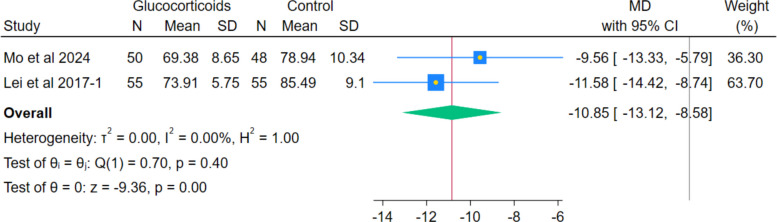
Forest plot illustrating the difference in Postoperative ICFS Score

The pooled analysis of postoperative Identity-Consequence Fatigue Scale (ICFS) revealed a statistically significant improvement in postoperative ICFS in the glucocorticoids group compared to the non-glucocorticoids group, with a pooled mean difference (MD) of − 10.85 (95% CI − 13.12 to − 8.58; *p* < 0.001; I^2^ = 0.00%). (Fig. 17) It should be noted that the ICFS is a general measure of fatigue, not a hip-specific functional score.

**Fig. 18:**
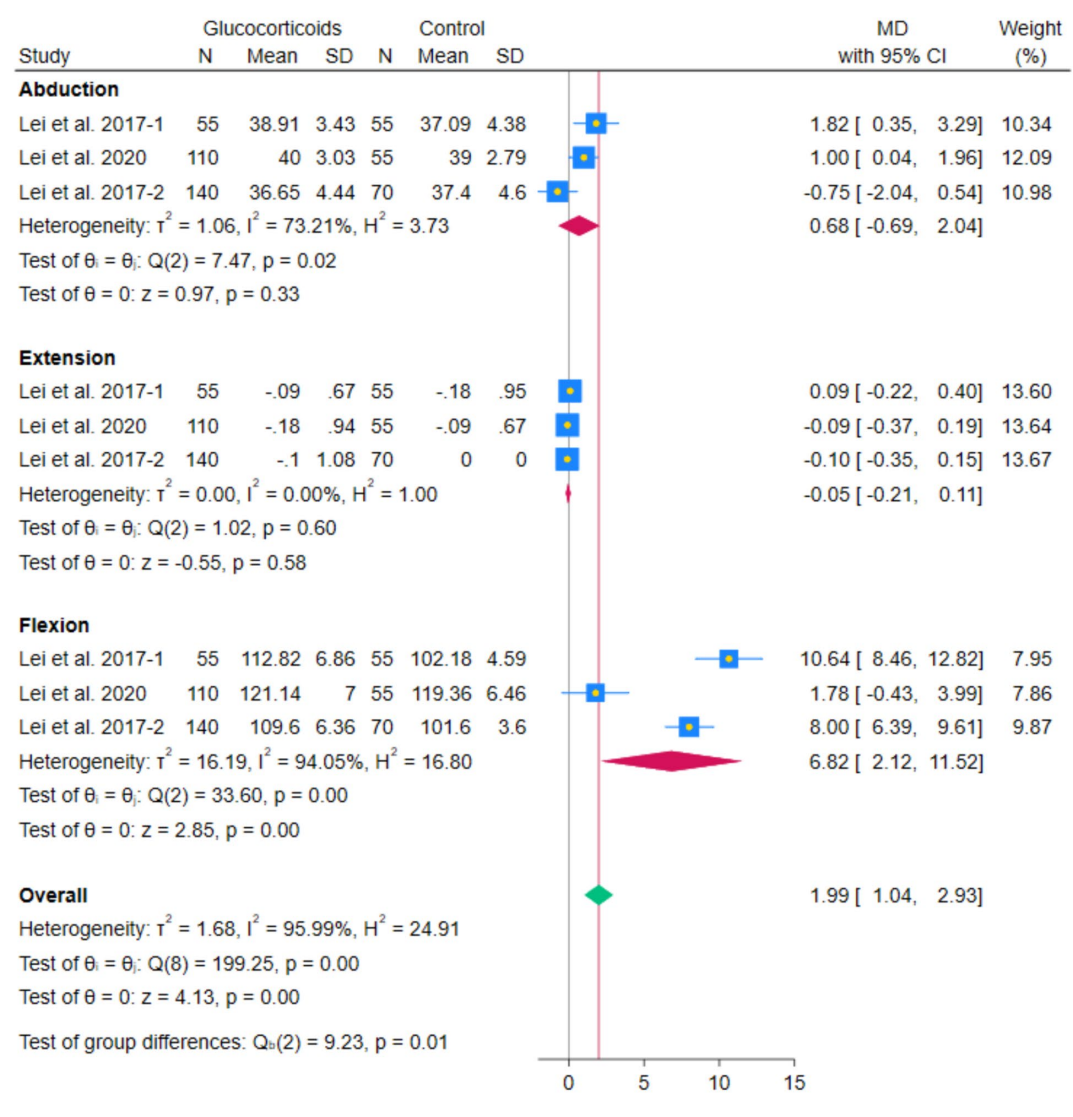
Forest plot illustrating the difference in postoperative abduction ROM, extension ROM, and flexion ROM

**Fig. 19:**
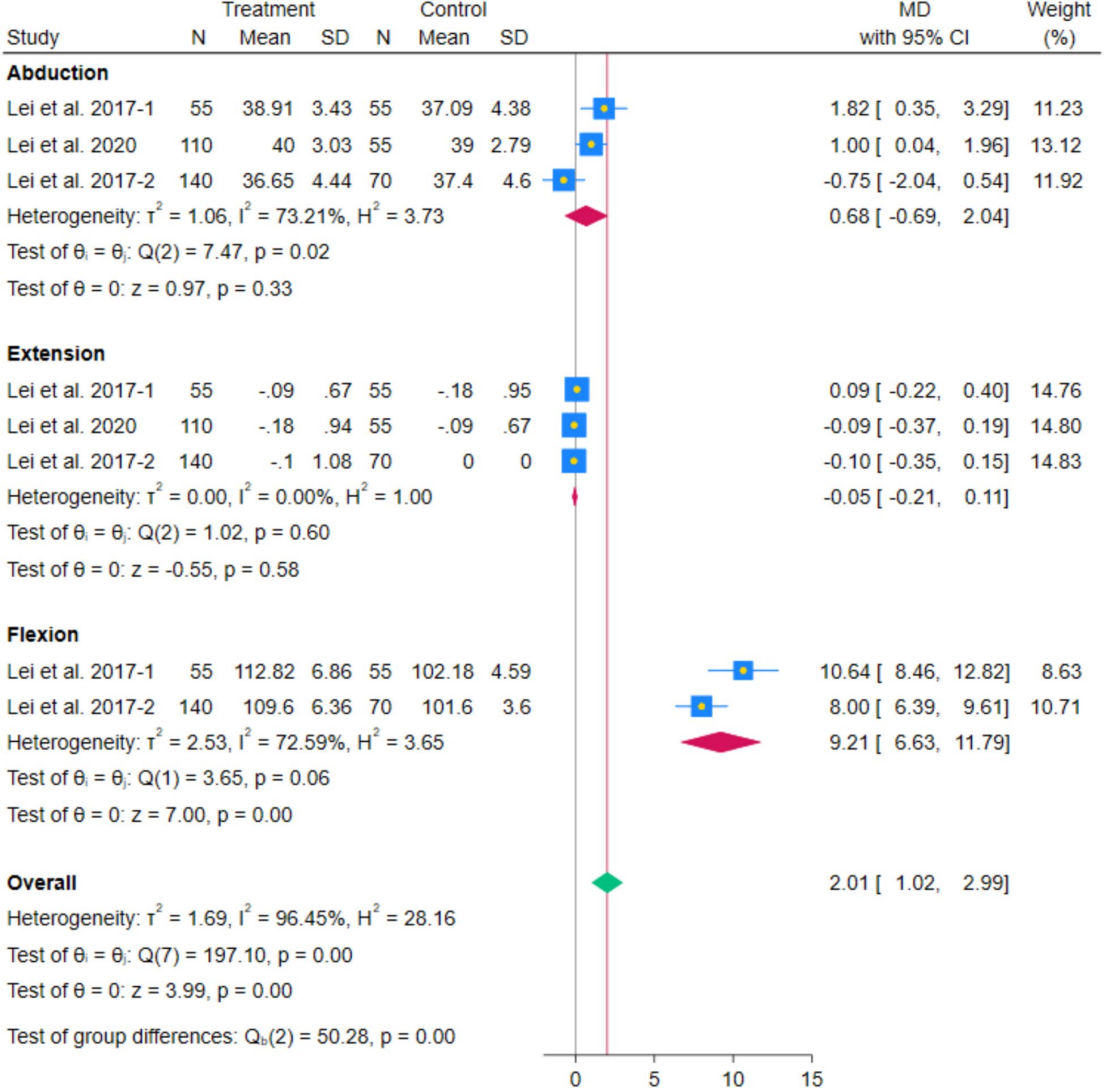
Forest plot illustrating the difference in postoperative abduction ROM, extension ROM, and flexion ROM after excluding Lei et al. 2020

The meta-analysis of 3 RCTs showed no statistically significant difference in abduction ROM between systemic glucocorticoids and controls (MD 0.68 degrees; 95% CI − 0.69 to 2.04; *p* = 0.33; I^2^ = 73.21%). Similarly, the pooled analysis of 3 RCTs showed no statistically significant difference in extension ROM between systemic glucocorticoids and controls (MD − 0.05 degrees; 95% CI − 0.21 to 0.11; *p* = 0.58; I^2^ = 0.00%). In contrast, the pooled analysis of 3 RCTs showed a statistically significant increase in flexion ROM between systemic glucocorticoids and controls (MD 6.82 degrees; 95% CI 2.12–11.52; *p* < 0.001; I^2^ = 94.05%). (Fig. 18) After excluding Lei et al. 2020, the increase remained statistically significant, and the heterogeneity decreased (MD 9.21 degrees; 95% CI 6.63–11.79; *p* < 0.001; I^2^ = 72.59%). (Fig. 19) These analyses, along with the limited number of included studies in the flexion ROM, highlight the fragility of this finding and the need for cautious interpretation. This pooled estimate should be interpreted as indicating a possible direction of benefit (increased flexion ROM) rather than a precise effect size.

### Secondary outcomes

The pooled analysis of 8 RCTs showed a statistically significant reduction in C-Reactive Protein (CRP) with systemic glucocorticoids compared to controls (MD − 57.50 mg/L; 95% CI − 71.45 to − 43.56; *p* < 0.001; I^2^ = 92.60%). (Supplementary Fig. S1) Leave-one-out sensitivity analyses remained statistically significant in all scenarios. Significant reductions were maintained on postoperative day 1 (MD − 27.94 mg/L; 95% CI − 37.30 to − 18.58; *p* < 0.001; I^2^ = 92.4%), day 2 (MD − 62.79 mg/L; 95% CI − 79.02 to − 46.56; *p* < 0.001; I^2^ = 93.83%), and day 3 (MD − 44.44 mg/L; 95% CI − 56.24 to − 32.64; *p* < 0.001; I^2^ = 83.92%). (Supplementary Fig. S2) Another subgroup analysis, stratified by glucocorticoid type and dose, demonstrated a significant reduction in CRP levels across all regimens. However, substantial heterogeneity persisted within each subgroup. (Supplementary Fig. S3) Due to very high statistical heterogeneity (I^2^ = 92.60%), this pooled estimate should be interpreted as indicating a consistent direction of effect (reduction in CRP with glucocorticoids) rather than a precise quantitative effect size.

The meta-analysis of 4 RCTs showed a statistically significant reduction in Interleukin-6 (IL-6) with systemic glucocorticoids (MD − 26.46 pg/mL; 95% CI − 32.36 to − 20.57; *p* < 0.001; I^2^ = 71.00%). (Supplementary Fig. S4) Leave-one-out sensitivity analyses remained statistically significant in all scenarios. Significant reductions were also consistent on postoperative day 1 (MD − 55.88 pg/mL; 95% CI − 75.00 to − 36.76; *p* < 0.001; I^2^ = 91.13%), day 2 (MD − 33.01 pg/mL; 95% CI − 54.16 to − 11.87; *p* < 0.001; I^2^ = 96.40%), and day 3 (MD − 25.80 pg/mL; 95% CI − 28.45 to − 23.15; *p* < 0.001; I^2^ = 0.00%). (Supplementary Fig. S5) Although heterogeneity was moderate to high (I^2^ = 71.00%), the consistent direction of effect (reduction in IL-6) across all 4 studies supports the robustness of this finding.

The pooled analysis revealed no statistically significant difference in the odds of gastrointestinal bleeding (OR 0.70, 95% CI 0.10–5.01; *p* = 0.72; I^2^ = 0.00%) (Supplementary Fig. S6), wound complications (OR 0.69, 95% CI 0.26–1.81; *p* = 0.45; I^2^ = 0.00%) (Supplementary Fig. S7), or infection rate (OR 0.66, 95% CI 0.17–2.53; *p* = 0.55; I^2^ = 0.00%) (Supplementary Fig. S8) between glucocorticoid and control groups. Leave-one-out sensitivity analyses remained non-significant in all scenarios.

A random-effects meta-analysis of 2 RCTs showed a statistically significant but mild increase in intraoperative blood loss with glucocorticoids (MD 61.17 mL, 95% CI 2.41–119.93; *p* = 0.04; I^2^ = 0.00%). (Supplementary Fig. S9) The odds of revision did not differ significantly between groups (OR 1.02, 95% CI 0.10–10.09; *p* = 0.99; I^2^ = 0.00%). (Supplementary Fig. S10).

The pooled analysis of 9 RCTs showed a statistically significant reduction in length of hospital stay (LOS) with systemic glucocorticoids compared to controls (MD − 0.46 days, 95% CI − 0.69 to − 0.22; *p* < 0.001; I^2^ = 70.60%). (Supplementary Fig. S11) Leave-one-out sensitivity analyses remained statistically significant in all scenarios.

The pooled analysis of 3 RCTs showed no statistically significant difference in operation time between groups (MD − 0.33 min, 95% CI − 11.49 to 10.82; *p* = 0.95; I^2^ = 78.03%). (Supplementary Fig. S12) After excluding Kardash et al. 2008, the result remained non-significant (MD 4.83 min, 95% CI − 2.28 to 11.95; *p* = 0.18; I^2^ = 48.36%). (Supplementary Fig. S13) Leave-one-out sensitivity analyses remained statistically non-significant in all cases.

## Discussion

Persistent pain and PONV often hinder recovery after total hip arthroplasty despite modern multimodal analgesia. Perioperative glucocorticoids, which reduce surgical stress, may help address these issues. However, optimal protocols and safety profiles—particularly regarding infection risk and glycemic control in diabetic patients—remain uncertain. Previous meta-analyses, such as that by Fan et al. [22], included only 3 trials. Moreover, recent meta-analyses have evaluated perioperative glucocorticoid use in broader total joint arthroplasty populations or have focused primarily on total knee arthroplasty (TKA), which limits procedure-specific inference for primary unilateral THA [23, 24]. To address this, we conducted a systematic review and meta-analysis of 12 RCTs (*n* = 1,128 patients) to evaluate the efficacy and safety of systemic glucocorticoids in primary unilateral total hip arthroplasty. Furthermore, we have evaluated the certainty of current evidence using the GRADE framework to raise the transparency of our conclusions.

Our analysis found that glucocorticoids revealed a modest reduction in VAS pain score during the first two days after surgery. At rest, pain levels dropped on postoperative day 1 (POD1) (MD − 0.30; *p* < 0.001) and POD2 (MD − 0.25; *p* = 0.03). Similarly, walking pain was reduced on POD1 (MD − 1.19; *p* < 0.001) and POD2 (MD − 0.54; *p* < 0.001). These findings are in line with a recent study on elderly THA patients that found pain relief from a single dose of dexamethasone [25]. They are consistent with broader meta-analytic findings showing early postoperative pain relief with glucocorticoids across various surgical areas [26]. When interpreted alongside the findings of Hannon et al. [23] and Liu et al. [24], the analgesic benefits observed in the present analysis appear consistent in direction but modest in magnitude. Importantly, both prior meta-analyses included broader arthroplasty populations and more heterogeneous risk profiles. In contrast, the current study specifically isolates primary unilateral THA and predominantly non-diabetic patients, which may partially explain the lower effect sizes observed. Notably, Lunn et al. observed significant pain reduction while walking with high-dose methylprednisolone (125 mg), suggesting that glucocorticoids are especially effective during physical activity, likely because they help reduce inflammation caused by movement [27].

However, statistical significance must be distinguished from clinical meaningfulness. Applying the minimal clinically important difference (MCID) threshold of − 1.86 cm on a 10-cm VAS scale established specifically for improving pain after THA by Danoff et al. [28], the observed POD1 walking pain reduction of − 1.19 cm falls substantially below this threshold. This indicates that even the most pronounced analgesic effect observed in our analysis—walking pain reduction on the first postoperative day—does not reach the level generally accepted as clinically perceptible to patients undergoing THA. All other pain reductions, at rest and on subsequent days, were considerably smaller and similarly below clinically meaningful thresholds. These findings suggest that while glucocorticoids produce statistically significant reductions in early postoperative pain, particularly during walking, the magnitude of effect is unlikely to be appreciated by patients as meaningful pain relief. The clinical relevance of this analgesic benefit may therefore lie not in direct pain perceptibility but in secondary effects such as reduced opioid consumption or enhanced mobilization, though these benefits were inconsistent in our analysis. Critically, the Danoff MCID was derived from pain measurements collected during the acute postoperative hospital stay (POD0–3), which aligns temporally with our primary outcome assessment window (POD1–3). This temporal alignment supports the appropriateness of applying this threshold to our early pain outcomes. However, we acknowledge two important caveats: first, the original MCID derivation did not separately estimate thresholds for pain at rest versus during walking; second, our application of a single threshold to both pain conditions assumes comparable responsiveness, which has not been formally validated. Despite these limitations, the substantial gap between our observed effects (MD − 1.19 cm) and the MCID threshold (− 1.86 cm) suggests that even under optimal assumptions, the analgesic benefit did not reach clinically perceptible levels.

Systemic glucocorticoids were similarly associated with a significant decrease in the overall occurrence of the composite PONV outcome (OR 0.21; *p* < 0.001), consistent with the known antiemetic properties of dexamethasone, as shown in previous reviews [29]. This was accompanied by a significant reduction in nausea severity measured by VAS 0–10 cm (MD − 0.68; *p* < 0.001) and the need for rescue antiemetics (OR 0.33; *p* < 0.001), which may support the robustness of the composite finding. These findings appear to be consistent with prior meta-analysis, which demonstrated a significant reduction in PONV following perioperative corticosteroid administration across total joint arthroplasty procedures, supporting their well-established antiemetic effect [23, 30]. Similarly, Liu et al. [24] reported a significant decrease in PONV incidence in patients receiving perioperative glucocorticoids for total knee arthroplasty (TKA), reinforcing the robustness and reproducibility of this effect across arthroplasty populations. Importantly, both meta-analyses identified PONV reduction as one of the most consistent and clinically relevant benefits of perioperative corticosteroid use. The present analysis supports these findings specifically in the context of primary unilateral THA, suggesting that the antiemetic benefit of glucocorticoids is preserved even when analyses are restricted to hip arthroplasty alone.

Opioid use showed inconsistent and unstable effects, with no significant reduction in morphine (MD − 8.75 mg; *p* = 0.09) and oxycodone (MD − 23.73 mg; *p* = 0.71). However, the reduction in morphine became statistically significant once Lei et al. 2020 was omitted, and the heterogeneity disappeared (MD − 5.29 mg; *p* = 0.04). This discrepancy likely reflects differences in administration and study protocols. Morphine was commonly delivered via patient-controlled analgesia (PCA), enabling patient-driven titration and potentially capturing subtle reductions in analgesic demand. In contrast, oxycodone was given orally on fixed or as-needed schedules, which may have obscured treatment effects. Differences in potency, route of administration (IV vs. oral), and baseline multimodal analgesia regimens—including the use of gabapentinoids or NSAIDs—may also have influenced outcomes. Additionally, morphine consumption was typically assessed within 24–48 h, while oxycodone use was measured over longer periods, possibly diluting early effects. These findings align with prior evidence suggesting protocol-dependent opioid-sparing effects and support the view that glucocorticoids function as adjunctive rather than primary opioid-sparing agents [23, 31].

Dosages and regimens varied widely across included studies (8–125 mg methylprednisolone equivalent), making it difficult to establish definitive dose recommendations and raising questions about the best regimen. For PONV prophylaxis alone, low-dose dexamethasone (4–8 mg) seems adequate [30]. However, our findings, along with those from Lunn et al. [28] and Yue et al. [32], suggest that higher doses may be needed to achieve broader anti-inflammatory effects and multimodal benefits. Consistent improvements in walking pain, flexion ROM, and inflammatory marker suppression were observed with dexamethasone 10 mg given in two doses [17, 19] or methylprednisolone 125 mg as a single dose [13–15, 18]. Lower doses used in combination treatments do not appear to reduce opioid use, highlighting that glucocorticoids should not be relied upon solely for pain relief and emphasizing the need for standardized protocols [31]. Balancing effectiveness, safety, and practicality, a single IV dose of dexamethasone 10–20 mg appears to be a reasonable candidate regimen for future prospective dose-finding RCTs. Such trials should stratify patients by diabetic status and include rigorous glycemic monitoring to assess safety in high-risk populations while remaining compatible with ERAS pathways.

Data on glycemic control were limited because 7 of 12 RCTs did not include patients with diabetes, focusing instead on non-diabetic or lower-risk groups. Postoperative glucose levels did not reveal any significant reductions (POD1: MD 0.00 mmol/L; *p* = 0.98; POD2: MD 0.06 mmol/L; *p* = 0.53; POD3: MD − 0.02 mmol/L; *p* = 0.88). Therefore, the apparent neutrality of glucocorticoids on glycemic outcomes observed in this meta-analysis applies strictly to non-diabetic populations and should not be extrapolated to diabetic patients. This contrasts with the findings of Liu et al. [24], who reported a statistically significant postoperative increase in blood glucose levels on POD1 following perioperative glucocorticoid administration. Furthermore, a recent meta-analysis of perioperative glucocorticoids in bilateral TKA reported a statistically significant increase in postoperative glucose levels (MD 22.19 mg/dL; *p* < 0.001), highlighting potential differences in glycemic impact across procedure type, surgical stress, and diabetic status within the included cohorts [27]. A significant retrospective study by Kebaish et al. [33] (*n* > 260,000 diabetic patients) offered additional proof of safety, even associating dexamethasone with lower rates of sepsis and periprosthetic joint infection (PJI). When combined with results from Williams et al. [34], who reported reduced LOS in diabetic patients, these data collectively may suggest, but do not prove, that glucocorticoids may be safely used even in high-risk populations, pending confirmation from prospective RCTs. However, because these external signals arise from non-randomized cohorts that are vulnerable to confounding and selection bias, the certainty of evidence for glycemic safety in diabetic THA populations remains very low. Therefore, until prospective RCT data are available, clinicians should consider glucose monitoring in diabetic patients receiving glucocorticoids, given the limited evidence in this group. Consequently, no conclusions regarding the safety or efficacy of perioperative glucocorticoids in diabetic THA patients can be drawn from the present meta-analysis.

Potential enhancements in functional recovery included a statistically significant increase in flexion range of motion (MD 6.82 degrees; *p* < 0.001) from three trials, though no significant improvements were observed for extension or abduction. This finding must be interpreted with caution due to the limited number of contributing studies and considerable statistical heterogeneity (I^2^ = 94.05%), likely reflecting differences in rehabilitation protocols, surgical practices, or assessment timing. Thus, the clinical relevance of this change remains uncertain. Patient-reported fatigue, measured by the Identity-Consequence Fatigue Scale (ICFS) score, also improved (MD − 10.85; *p* < 0.001), though this tool is not a standard measure for hip-specific function. While improvements in ROM may promote early mobilization, their clinical significance requires confirmation in larger, more homogeneous studies. While these directional findings are consistent with earlier research linking perioperative steroids to improved early mobility [28, 35, 36], their clinical significance is unclear.

The safety profile appears to be reassuring, with no observed increase in measured complications, including surgical revisions (OR 1.02; *p* = 0.99), gastrointestinal bleeding (OR 0.70; *p* = 0.72), wound complications (OR 0.69; *p* = 0.45), or infection rates (OR 0.66; *p* = 0.55), all with low heterogeneity (I^2^ = 0%). However, these findings must be interpreted in the context of the included studies, which predominantly involved lower-risk, non-diabetic patients and had limited follow-up to detect rare adverse events. There was a slight increase in blood loss during surgery (MD 61.17 mL; *p* = 0.04), suggesting the intraoperative difference is unlikely to be clinically relevant in the context of typical THA blood loss volumes [37]. These results align with high-quality randomized controlled trials [25] and previous meta-analyses [26]. A large retrospective cohort by Mou et al. [38] found no association between perioperative low- or high-dose dexamethasone and hospital readmission, infection, or wound complications in primary total joint arthroplasty, including THA. Moreover, Richardson et al. [39] also showed no significant increase in periprosthetic joint infection (PJI) incidence after total knee and hip arthroplasties with low-dose dexamethasone. Similarly, Yoshida et al. [40] reported reduced risks of acute kidney injury and urinary tract infections, suggesting protective effects on multiple organs. Glucocorticoids showed a potential reduction in CRP levels on POD1 (MD − 27.94 mg/L; *p* < 0.001), POD2 (MD − 62.79 mg/L; *p* < 0.001), and POD3 (MD − 44.44 mg/L; *p* < 0.001). The pooled subgroup analysis by dose and type similarly showed a significant reduction in CRP levels: Dexamethasone > 20 mg (MD − 114.59 mg/L; *p* < 0.001), Dexamethasone ≤ 20 mg (MD − 40.34 mg/L; *p* < 0.001), and Methylprednisolone 125 mg (MD − 52.77 mg/L; *p* = 0.01). IL-6 also fell on POD1 (MD − 55.88 pg/mL; *p* < 0.001), POD2 (MD − 33.01 pg/mL; *p* < 0.001), and POD3 (MD − 25.80 pg/mL; *p* < 0.001). These decreases may correspond with clinical improvements in pain and PONV, although they are affected by considerable heterogeneity, so this observed association should be interpreted cautiously. Glucocorticoids were also associated with a reduction in the length of hospital stay (LOS) (MD − 0.46 days; *p* < 0.001). The observed reduction in LOS is further supported by extensive observational data; for example, Yoshida et al. [39] (*n* ≈ 200,000 patients) reported a mean LOS reduction of 0.37 days with dexamethasone use.

Substantial statistical heterogeneity was observed for several key outcomes, including inflammatory markers (CRP: I^2^ = 92.60%; IL-6: I^2^ = 71.00%) and flexion range of motion (I^2^ = 94.05%). Consistent with our pre-specified analytic framework, these pooled estimates should be interpreted as indicating a direction of effect rather than precise effect sizes. Drawing directly from study-level data, we identified multiple sources of heterogeneity, with glucocorticoid regimen variability being the most prominent. To help navigate this complexity and understand which specific regimens drive the observed signals, we constructed an evidence map that synthesizes outcomes by glucocorticoid type, dose, and schedule. Several patterns emerge from this map despite the limitations of indirect comparison. First, walking pain reduction—most pronounced on POD1 (MD − 1.19)—was consistently observed with multiple-dose dexamethasone regimens [17, 19]. Second, PONV reduction demonstrated remarkable consistency across all regimens (OR 0.21), supporting a class effect achievable even with low-dose dexamethasone and consistent with established antiemetic literature [30]. Third, flexion ROM improvement (MD 6.82 degrees) was driven exclusively by multiple-dose dexamethasone studies [17, 19], suggesting sustained glucocorticoid exposure may be necessary for functional benefits. Fourth, glycemic safety data remain almost derived from high-dose methylprednisolone studies in non-diabetic populations, highlighting a critical evidence gap for lower-intensity regimens and diabetic patients. These hypothesis-generating observations require confirmation in head-to-head trials but provide a framework for moving beyond the question “do glucocorticoids work?" to the more clinically relevant question “which regimen works best for which outcome?”.

### Clinical guidance and future directions

The findings of this meta-analysis should be interpreted as hypothesis-generating rather than as a basis for definitive clinical recommendations. The analysis suggests potential benefits in reducing early pain and PONV incidence for patients undergoing primary unilateral THA. However, the integration of glucocorticoids into clinical practice requires careful consideration of the available evidence. The majority of included studies excluded patients with diabetes mellitus, meaning the safety profile of systemic glucocorticoids—particularly regarding glycemic control—is not established for this high-risk population, and their routine use cannot be recommended without further targeted RCTs. The observed reductions in inflammatory biomarkers, though statistically significant, were associated with very high heterogeneity, reflecting variability in surgical stress, dosing, and measurement protocols. Crucially, the optimal dose, agent, and timing remain undefined due to significant variation across trials (8–125 mg methylprednisolone equivalent); we were unable to perform a formal dose–response analysis from the available data. Based on our evidence map, a single intravenous dose of dexamethasone 10–20 mg represents a reasonable candidate regimen for investigation in future prospective trials, capturing the most effective elements from both multiple-dose dexamethasone and high-dose methylprednisolone protocols while maintaining practical single-administration suitability for ERAS pathways. Therefore, if glucocorticoids are considered within an institutional protocol, they should be viewed as one component of a multimodal analgesia and antiemetic strategy, not a standalone solution. Glucose monitoring is recommended, especially in patients with or at risk for diabetes. The modest reduction in LOS and potential improvement in early flexion range of motion (ROM) may support the direction of faster functional recovery within enhanced recovery pathways, but these findings require validation in studies with standardized rehabilitation protocols. In summary, these results may support the rationale for including glucocorticoids in future, well-designed RCTs aimed at optimizing perioperative pathways, but do not justify a universal change in practice.

Future RCTs should determine optimal doses in dexamethasone-equivalent ranges to identify the minimal effective dose for pain relief and PONV reduction while maintaining glycemic control. Based on our evidence map, a single intravenous dose of dexamethasone 10–20 mg represents a reasonable candidate regimen for investigation, capturing the most effective elements from both multiple-dose dexamethasone and high-dose methylprednisolone protocols while maintaining practical single-administration suitability for ERAS pathways. There is a need for standardized definitions and timing for PONV components, as well as unified patient-reported outcome measures with sufficient follow-up to track functional improvements. Well-designed randomized trials that include diabetic patients and categorize them based on their initial glycemic control are crucial to verify safety and effectiveness in this high-risk population. Additionally, comparative studies of different types of glucocorticoids and their administration timing, along with practical assessments integrated into standardized Enhanced Recovery After Surgery (ERAS) pathways, will help clarify the effects of protocols on clinical outcomes of primary unilateral THA.

### Strengths and limitations

This meta-analysis has several key strengths. First, it focuses exclusively on primary unilateral total hip arthroplasty (THA) and includes 12 randomized controlled trials (RCTs), ensuring a homogeneous and high-quality evidence base. Methodological rigor was upheld through the use of the Cochrane RoB 2 tool for risk of bias assessment, including domain-level evaluations, and adherence to PRISMA guidelines. The certainty of evidence was graded using the GRADE approach, revealing moderate certainty for VAS pain scores at rest and during motion, and low certainty for postoperative glucose levels and infection rates. Clinically, the analysis provides a comprehensive evaluation of multiple outcomes—such as pain at rest and with movement, PONV composites and severity, opioid types, range of motion, patient-reported function, inflammatory markers, glycemia, length of stay, and adverse events—thereby enhancing the clinical significance of the findings. However, several important limitations must be acknowledged. First, our search was restricted to English-language publications and randomized controlled trials (RCTs), a choice made to prioritize internal validity. However, this inevitably introduces the potential for selection, publication, and language bias by excluding relevant data from non-English sources and grey literature. This limitation suggests that positive results are more likely to be published in English-language journals, potentially leading to an overestimation of reported outcomes. The generalizability of the findings is constrained, as seven of the twelve included RCTs explicitly excluded patients with diabetes mellitus, meaning the efficacy and safety profile—particularly for glycemic control—is not established for this clinically important population. Moreover, the potential inclusion of undiagnosed or borderline diabetic patients represents an additional limitation when interpreting the glycemic safety profile. Substantial statistical heterogeneity was observed for several key outcomes, including inflammatory markers (CRP; I^2^ = 92.60%, IL-6; I^2^ = 71.00%) and flexion range of motion (I^2^ = 94.05%), which, despite sensitivity analyses, limits the precision of the pooled estimates and reflects underlying clinical and methodological diversity in surgical technique, rehabilitation, and glucocorticoid regimens. Critically, this heterogeneity directly affects the strength of our conclusions: for outcomes with I^2^ > 75%, the pooled effect estimates should be interpreted as indicating a direction of benefit rather than providing precise effect sizes suitable for clinical decision-making. Importantly, the Danoff MCID was derived from acute postoperative pain measurements during the hospital stay (POD0–3), making it temporally appropriate for our analysis; however, we acknowledge that the original MCID derivation did not separately estimate thresholds for pain at rest versus during walking. Our application of a single MCID threshold to both pain conditions assumes comparable responsiveness, which has not been formally validated. Future studies should derive activity-specific MCIDs for acute pain after THA. Furthermore, the wide variation in glucocorticoid types and doses (8–125 mg methylprednisolone equivalent) across studies precluded a meaningful exploration of dose–response relationships, a critical gap for clinical implementation. The follow-up duration in most studies was limited to the early postoperative period, preventing assessment of long-term complications and functional outcomes. Variability in concomitant analgesic and antiemetic protocols across trials may have confounded the isolated effect of glucocorticoids on outcomes like opioid consumption. Finally, the reliability of the evidence is tempered by the fact that four of the twelve included studies were judged to have a high overall risk of bias.

## Conclusion

In conclusion, this meta-analysis suggests—but does not definitively establish—that perioperative systemic glucocorticoids in primary unilateral THA may modestly reduce early pain, with the maximal reduction observed on pod1 during walking. However, these reductions did not reach the MCID threshold, and their clinical value remains uncertain. They similarly were associated with reductions in PONV severity and incidence, rescue antiemetic use, inflammatory biomarkers, and length of hospital stay, without evidence of increased short-term wound complications or infections in predominantly non-diabetic populations. Because most included RCTs excluded patients with diabetes, these findings cannot be extrapolated to diabetic patients, and the observed glycemic neutrality applies only to non-diabetic cohorts. A potential benefit for early postoperative flexion ROM was noted, but it is based on limited, highly heterogeneous data. A single IV dose of dexamethasone 10–20 mg emerges as a reasonable candidate for investigation by future trials. Overall, these findings provide hypothesis-generating evidence to guide further prospective RCTs aimed at optimizing dosing, evaluating high-risk populations, and integrating glucocorticoids within ERAS pathways for total hip arthroplasty.

## Supplementary Information

Supplementary Material 1.

## Data Availability

All data generated or analyzed during this study are included in the published article and its supplementary materials.

## Abbreviations

ASA: American Society of Anesthesiologists
CI: Confidence Interval
CRP: C-reactive Protein
ERAS: Enhanced Recovery After Surgery
GRADE: Grading of Recommendations Assessment, Development and Evaluation
ICFS: Identity-Consequence Fatigue Scale
IL-6: Interleukin-6
IV: Intravenous
LOS: Length of Stay
MD: Mean Difference
NR: Not reported
OR: Odds Ratio
PJI: Periprosthetic Joint Infection
POD1: Postoperative Day 1
PONV: Postoperative Nausea and Vomiting
PRISMA: Preferred Reporting Items for Systematic Reviews and Meta-Analyses
RCT: Randomized Controlled Trial
RoB 2: Risk of Bias 2
ROM: Range of Motion
SD: Standard Deviation
THA: Total Hip Arthroplasty
TJA: Total Joint Arthroplasty
TKA: Total Knee Arthroplasty
VAS: Visual analogue scale
