# Supplementary material for: Comparative clinical efficacy and safety of perioperative systemic glucocorticoids in primary unilateral total hip arthroplasty: a GRADE-assessed meta-analysis of randomized controlled trials
Source: Arthroplasty. 2026 Jun 2;8:40. doi: 10.1186/s42836-026-00397-4 (PMC13227744; doi:10.1186/s42836-026-00397-4)
Supplement: Supplementary file 1 — Supplementary Material 1. [file 42836_2026_397_MOESM1_ESM.docx]

**Supplementary File**

**List of contents:**

1. Tables

**Supplementary Table S1**. The full search strings, including Boolean operators.

**Supplementary Table S2**. PRISMA 2020 main checklist.

1. Figures:

**Supplementary Fig. S1**. Forest plot illustrating the difference in C-reactive protein (CRP) levels at the last reported time point.

**Supplementary Fig. S2**. Forest plot illustrating the subgroup analysis of C-reactive protein (CRP) levels at different follow-up intervals (POD1–3).

**Supplementary Fig. S3**. Forest plot illustrating the subgroup analysis of C-reactive protein (CRP) levels according to the glucocorticoid agent at the last follow-up.

**Supplementary Fig. S4.** Forest plot illustrating the difference in Interleukin-6 (IL-6) levels at the last reported time point.

**Supplementary Fig. S5**. Forest plot illustrating the subgroup analysis of Interleukin-6 (IL-6) levels at different follow-up intervals (POD1–3).

**Supplementary Fig. S6**. Forest plot illustrating the incidence of postoperative gastrointestinal bleeding.

**Supplementary Fig. S7.** Forest plot illustrating the incidence of postoperative wound complications.

**Supplementary Fig. S8**. Forest plot illustrating the incidence of postoperative infection.

**Supplementary Fig. S9**. Forest plot illustrating the difference in intraoperative blood loss.

**Supplementary Fig. S10.** Forest plot illustrating the incidence of revision procedures.

**Supplementary Fig. S11**. Forest plot illustrating the mean difference in length of hospital stay (LOS).

**Supplementary Fig. S12**. Forest plot illustrating the difference in operative time.

**Supplementary Fig. S13.** Forest plot illustrating the difference in operative time after excluding Kardash et al. 2008.

**Supplementary Fig. S14.** Leave-one-out analysis for the VAS pain at rest sensitivity analysis outcome, showing the change in the pooled effect after removing each study.

**Supplementary Fig. S15**. Leave-one-out analysis for the VAS pain during walk sensitivity analysis outcome, showing the change in the pooled effect after removing each study.

**Supplementary Fig. S16**. Leave-one-out analysis for the blood glucose level sensitivity analysis outcome, showing the change in the pooled effect after removing each study.

**Supplementary Fig. S17.** Leave-one-out analysis for the VAS-Nausea sensitivity analysis outcome, showing the change in the pooled effect after removing each study.

**Supplementary Fig. S18.** Leave-one-out analysis for the PONV sensitivity analysis outcome, showing the change in the pooled effect after removing each study.

**Supplementary Fig. S19**. Leave-one-out analysis for the rescue antiemetic use sensitivity analysis outcome, showing the change in the pooled effect after removing each study.

**Supplementary Fig. S20.** Leave-one-out analysis for the CRP sensitivity analysis outcome, showing the change in the pooled effect after removing each study.

**Supplementary Fig. S21.** Leave-one-out analysis for the IL-6 sensitivity analysis outcome, showing the change in the pooled effect after removing each study.

**Supplementary Fig. S22.** Leave-one-out analysis for the length of stay sensitivity analysis outcome, showing the change in the pooled effect after removing each study.

**Supplementary Fig. S23.** Leave-one-out analysis for the gastrointestinal bleeding sensitivity analysis outcome, showing the change in the pooled effect after removing each study.

**Supplementary Fig. S24.** Leave-one-out analysis for the infection rate sensitivity analysis outcome, showing the change in the pooled effect after removing each study.

**Supplementary Fig. S25**. Leave-one-out analysis for the wound complications’ sensitivity analysis outcome, showing the change in the pooled effect after removing each study.

**Supplementary Fig. S26**. Leave-one-out analysis for the operative time sensitivity analysis outcome, showing the change in the pooled effect after removing each study.

**Supplementary Table S1.** The full search strings, including Boolean operators

| **Database** | **Search strings** | **Number of outcomes** | **Date of search** |
| --- | --- | --- | --- |
| PubMed | (("total hip arthroplast*"[tiab] OR "total hip replacement"[tiab] OR "THA"[tiab] OR "THR"[tiab] OR "hip arthroplasty"[tiab] OR "hip replacement"[tiab] OR "unilateral total hip arthroplasty"[tiab] OR "primary unilateral total hip arthroplasty"[tiab] OR "unilateral hip replacement"[tiab] OR "unilateral THA"[tiab] OR "Arthroplasty, Replacement, hip"[Mesh]) AND ("Glucocorticoid*"[Mesh] OR "Adrenal Cortex Hormones"[Mesh] OR "Cortisone"[Mesh] OR "Dexamethasone"[Mesh] OR "Betamethasone"[Mesh] OR "Methylprednisolone"[Mesh] OR "Hydrocortisone"[Mesh] OR "Triamcinolone"[Mesh] OR "Prednisone"[Mesh] OR "Prednisolone"[Mesh] OR "Cortisone"[tiab] OR "Dexamethasone"[tiab] OR "Betamethasone"[tiab] OR "Methylprednisolone"[tiab] OR "Hydrocortisone"[tiab] OR "Triamcinolone"[tiab] OR "Prednisone"[tiab] OR "Prednisolone"[tiab] OR "steroid*"[tiab] OR "corticosteroid*"[tiab])) | 1122 | 17/10/2025 |
| Scopus | TITLE-ABS-KEY(((“total hip arthroplast*” OR “total hip replacement” OR “THA” OR “THR” OR “hip arthroplasty” OR “hip replacement” OR “unilateral total hip arthroplasty” OR “primary unilateral total hip arthroplasty" OR “unilateral hip replacement" OR “unilateral THA” OR “Arthroplasty, Replacement, Hip”) AND (“Glucocorticoid*” OR “Adrenal Cortex Hormones" OR “Cortisone” OR “Dexamethasone” OR “Betamethasone” OR “Methylprednisolone” OR “Hydrocortisone” OR “Triamcinolone” OR “Prednisone” OR “Prednisolone” OR “steroid*” OR “corticosteroid*”))) | 2708 | 17/10/2025 |
| Web of Science (WOS) | TS=((("total hip arthroplast*" OR “total hip replacement" OR ”THA” OR “THR” OR “hip arthroplasty” OR “hip replacement” OR “unilateral total hip arthroplasty” OR “primary unilateral total hip arthroplasty” OR “unilateral hip replacement” OR “unilateral THA” OR “Arthroplasty, Replacement, Hip”) AND (“Glucocorticoid*” OR “Adrenal Cortex Hormones" OR ”Cortisone” OR “Dexamethasone” OR “Betamethasone” OR “Methylprednisolone” OR “Hydrocortisone” OR “Triamcinolone” OR “Prednisone” OR “Prednisolone” OR “steroid*” OR “corticosteroid*”))) | 1033 | 17/10/2025 |
| Cochrane Library | (“total hip arthroplast*" OR ”total hip replacement" OR ”THA” OR “THR” OR “hip arthroplasty” OR “hip replacement” OR “unilateral total hip arthroplasty” OR “primary unilateral total hip arthroplasty” OR “unilateral hip replacement” OR “unilateral THA” OR “Arthroplasty Replacement Hip”):ti,ab,kw AND (“Glucocorticoid*” OR “Adrenal Cortex Hormones" OR ”Cortisone” OR “Dexamethasone” OR “Betamethasone” OR “Methylprednisolone” OR “Hydrocortisone” OR “Triamcinolone” OR “Prednisone” OR “Prednisolone” OR “steroid*” OR “corticosteroid*”) | 419 | 17/10/2025 |

**Supplementary Table S2.** PRISMA 2020 main checklist

**PRISMA 2020 main checklist**

| **Topic** | **No.** | **Item** | **Location where item is reported** |
| --- | --- | --- | --- |
| **TITLE** |  |  |  |
| **Title** | 1 | Identify the report as a systematic review. | Title Page |
| **ABSTRACT** |  |  |  |
| **Abstract** | 2 | See the PRISMA 2020 for Abstracts checklist |  |
| **INTRODUCTION** |  |  |  |
| **Rationale** | 3 | Describe the rationale for the review in the context of existing knowledge. | Introduction - 3^rd^ paragraph |
| **Objectives** | 4 | Provide an explicit statement of the objective(s) or question(s) the review addresses. | Introduction - 3^rd^ paragraph |
| **METHODS** |  |  |  |
| **Eligibility criteria** | 5 | Specify the inclusion and exclusion criteria for the review and how studies were grouped for the syntheses. | Methods – Inclusion & Exclusion Criteria section |
| **Information sources** | 6 | Specify all databases, registers, websites, organisations, reference lists, and other sources searched or consulted to identify studies. Specify the date when each source was last searched or consulted. | Methods – Search Strategy Section |
| **Search strategy** | 7 | Present the full search strategies for all databases, registers, and websites, including any filters and limits used. | In the Supplementary Materials File 1 |
| **Selection process** | 8 | Specify the methods used to decide whether a study met the inclusion criteria of the review, including how many reviewers screened each record and each report retrieved, whether they worked independently, and, if applicable, details of automation tools used in the process. | Methods – Inclusion & Exclusion Criteria section |
| **Data collection process** | 9 | Specify the methods used to collect data from reports, including how many reviewers collected data from each report, whether they worked independently, any processes for obtaining or confirming data from study investigators, and, if applicable, details of automation tools used in the process. | Methods – Data Extraction section |
| **Data items** | 10a | List and define all outcomes for which data were sought. Specify whether all results that were compatible with each outcome domain in each study were sought (e.g., for all measures, time points, analyses), and if not, the methods used to decide which results to collect. | Methods – Data Extraction section |
|  | 10b | List and define all other variables for which data were sought (e.g., participant and intervention characteristics, funding sources). Describe any assumptions made about any missing or unclear information. | Methods – Data Extraction section |
| **Study risk of bias assessment** | 11 | Specify the methods used to assess risk of bias in the included studies, including details of the tool(s) used, how many reviewers assessed each study, and whether they worked independently, and if applicable, details of automation tools used in the process. | Methods – ROB section |
| **Effect measures** | 12 | Specify for each outcome the effect measure(s) (e.g., risk ratio, mean difference) used in the synthesis or presentation of results. | Methods – Data Synthesis section |
| **Synthesis methods** | 13a | Describe the processes used to decide which studies were eligible for each synthesis (e.g., tabulating the study intervention characteristics and comparing against the planned groups for each synthesis (item 5)). | Methods – Data Synthesis section |
|  | 13b | Describe any methods required to prepare the data for presentation or synthesis, such as handling of missing summary statistics or data conversions. | Methods – Data Synthesis section |
|  | 13c | Describe any methods used to tabulate or visually display the results of individual studies and syntheses. | Methods – Data Synthesis section |
|  | 13d | Describe any methods used to synthesize results and provide a rationale for the choice(s). If meta-analysis was performed, describe the model(s), method(s) to identify the presence and extent of statistical heterogeneity, and software package(s) used. | Methods – Data Synthesis section |
|  | 13e | Describe any methods used to explore possible causes of heterogeneity among study results (e.g., subgroup analysis, meta-regression). | Methods – Data Synthesis section |
|  | 13f | Describe any sensitivity analyses conducted to assess the robustness of the synthesized results. | Methods – Data Synthesis section |
| **Reporting bias assessment** | 14 | Describe any methods used to assess the risk of bias due to missing results in a synthesis (arising from reporting biases). | Methods – Data Synthesis section |
| **Certainty assessment** | 15 | Describe any methods used to assess certainty (or confidence) in the body of evidence for an outcome. | Methods |
| **RESULTS** |  |  |  |
| **Study selection** | 16a | Describe the results of the search and selection process, from the number of records identified in the search to the number of studies included in the review, ideally using a flow diagram. | Results – Screening Process & Study Selection |
|  | 16b | Cite studies that might appear to meet the inclusion criteria, but which were excluded, and explain why they were excluded. | In PRISMA flowchart |
| **Study characteristics** | 17 | Cite each included study and present its characteristics. | Results – Tables |
| **Risk of bias in studies** | 18 | Present assessments of risk of bias for each included study. | Results – ROB Assessment |
| **Results of individual studies** | 19 | For all outcomes, present, for each study: (a) summary statistics for each group (where appropriate) and (b) an effect estimate and its precision (e.g., confidence/credible interval), ideally using structured tables or plots. | Results |
| **Results of syntheses** | 20a | For each synthesis, briefly summarise the characteristics and risk of bias among contributing studies. | Discussion – Limitations |
|  | 20b | Present the results of all statistical syntheses conducted. If meta-analysis was done, present for each the summary estimate and its precision (e.g., confidence/credible interval) and measures of statistical heterogeneity. If comparing groups, describe the direction of the effect. | Results |
|  | 20c | Present the results of all investigations of possible causes of heterogeneity among study results. | Results |
|  | 20d | Present the results of all sensitivity analyses conducted to assess the robustness of the synthesized results. | Results |
| **Reporting biases** | 21 | Present assessments of risk of bias due to missing results (arising from reporting biases) for each synthesis assessed. | N/A |
| **Certainty of evidence** | 22 | Present assessments of certainty (or confidence) in the body of evidence for each outcome assessed. | N/A |
| **DISCUSSION** |  |  |  |
| **Discussion** | 23a | Provide a general interpretation of the results in the context of other evidence. | Discussion |
|  | 23b | Discuss any limitations of the evidence included in the review. | Discussion – Limitations section |
|  | 23c | Discuss any limitations of the review processes used. | Discussion – Limitations section |
|  | 23d | Discuss implications of the results for practice, policy, and future research. | Discussion – Implications and Future Directions |
| **OTHER INFORMATION** |  |  |  |
| **Registration and protocol** | 24a | Provide registration information for the review, including register name and registration number, or state that the review was not registered. | Methods – Search Strategy Section |
|  | 24b | Indicate where the review protocol can be accessed, or state that a protocol was not prepared. | Methods – Search Strategy Section |
|  | 24c | Describe and explain any amendments to information provided at registration or in the protocol. | Methods – Search Strategy Section |
| **Support** | 25 | Describe sources of financial or non-financial support for the review, and the role of the funders or sponsors in the review. | Declarations – Funding statement |
| **Competing interests** | 26 | Declare any competing interests of review authors. | Declarations – Competing Interest |
| **Availability of data, code, and other materials** | 27 | Report which of the following are publicly available and where they can be found: template data collection forms; data extracted from included studies; data used for all analyses; analytic code; any other materials used in the review. | Declarations – Availability of Data and Materials |

**PRIMSA abstract checklist**

| **Topic** | **No.** | **Item** | **Reported?** |
| --- | --- | --- | --- |
| **TITLE** |  |  |  |
| **Title** | 1 | Identify the report as a systematic review. | Yes |
| **BACKGROUND** |  |  |  |
| **Objectives** | 2 | Provide an explicit statement of the main objective(s) or question(s) the review addresses. | Yes |
| **METHODS** |  |  |  |
| **Eligibility criteria** | 3 | Specify the inclusion and exclusion criteria for the review. | Yes |
| **Information sources** | 4 | Specify the information sources (e.g., databases, registers) used to identify studies and the date when each was last searched. | Yes |
| **Risk of bias** | 5 | Specify the methods used to assess risk of bias in the included studies. | Yes |
| **Synthesis of results** | 6 | Specify the methods used to present and synthesize results. | Yes |
| **RESULTS** |  |  |  |
| **Included studies** | 7 | Give the total number of included studies and participants, and summarise relevant characteristics of studies. | Yes |
| **Synthesis of results** | 8 | Present results for main outcomes, preferably indicating the number of included studies and participants for each. If a meta-analysis was done, report the summary estimate and confidence/credible interval. If comparing groups, indicate the direction of the effect (i.e., which group is favoured). | Yes |
| **DISCUSSION** |  |  |  |
| **Limitations of evidence** | 9 | Provide a summary of the limitations of the evidence included in the review (e.g., study risk of bias, inconsistency, and imprecision). | Yes |
| **Interpretation** | 10 | Provide a general interpretation of the results and important implications. | Yes |
| **OTHER** |  |  |  |
| **Funding** | 11 | Specify the primary source of funding for the review. | No, as it was provided in the decelerations section |
| **Registration** | 12 | Provide the register name and registration number. | Yes |

*From:* Page MJ, McKenzie JE, Bossuyt PM, Boutron I, Hoffmann TC, Mulrow CD, et al. The PRISMA 2020 statement: an updated guideline for reporting systematic reviews. MetaArXiv. 2020, September 14. DOI: 10.31222/osf.io/v7gm2. For more information, visit: [www.prisma-statement.org](file:///C:\Users\original%20store\Downloads\Telegram%20Desktop\www.prisma-statement.org)


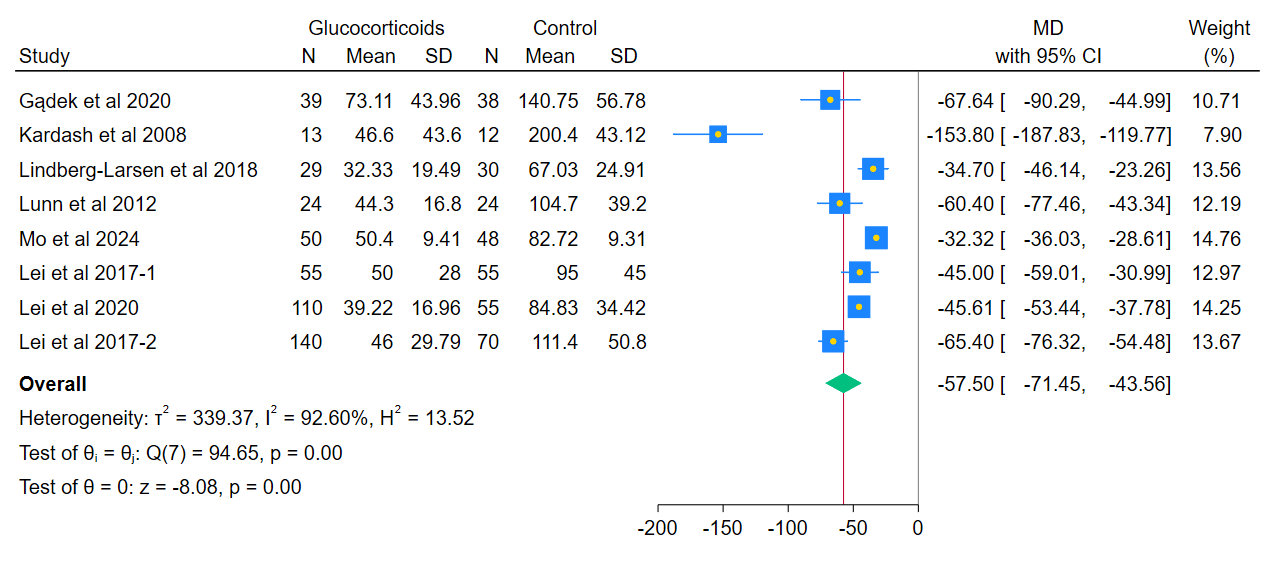


**Supplementary Fig. S1.** Forest plot illustrating the difference in C-reactive protein (CRP) levels at the last reported time point.


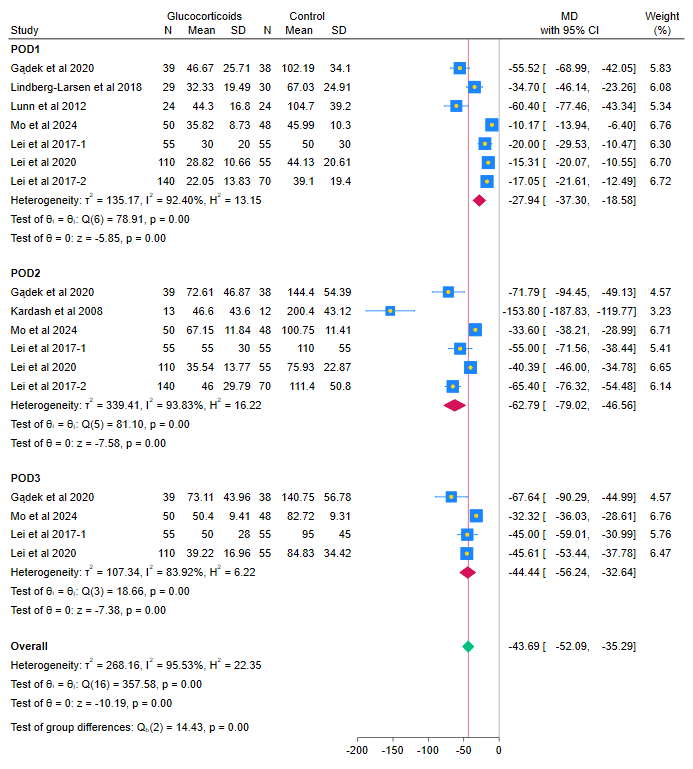


**Supplementary Fig. S2.** Forest plot illustrating the subgroup analysis of C-reactive protein (CRP) levels at different follow-up intervals (POD1–3).

**
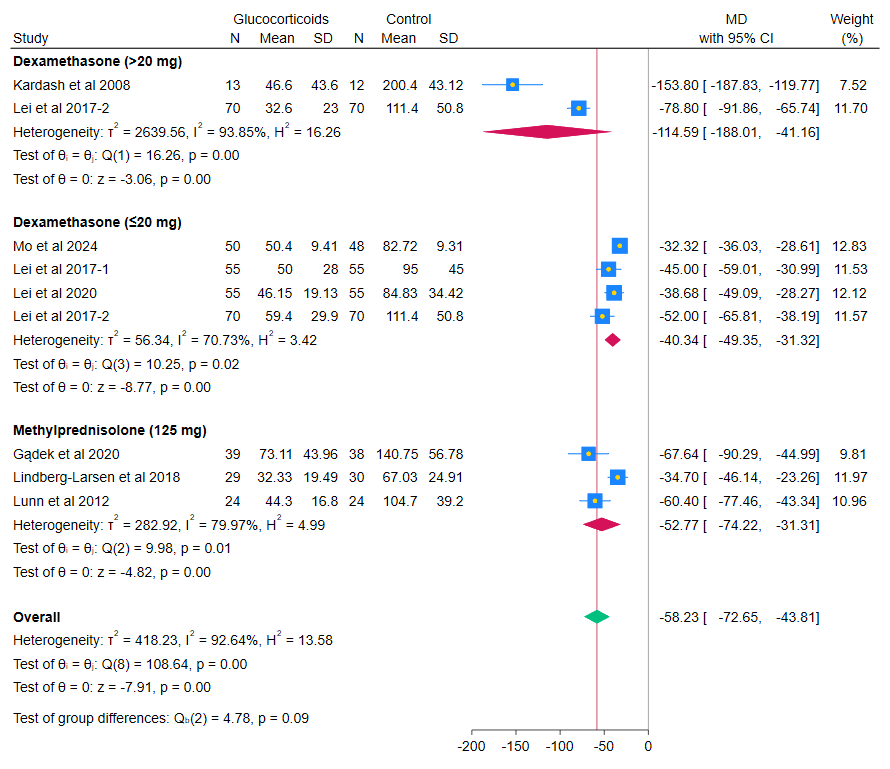
**

**Supplementary Fig. S3.** Forest plot illustrating the subgroup analysis of C-reactive protein (CRP) levels according to the glucocorticoid agent at the last follow-up.

**
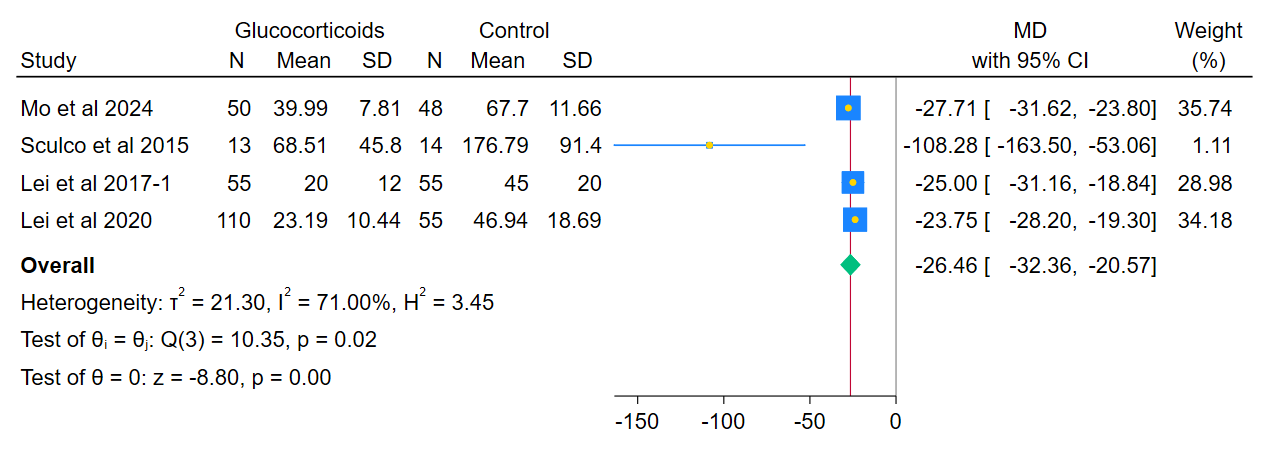
**

**Supplementary Fig. S4.** Forest plot illustrating the difference in Interleukin-6 (IL-6) levels at the last reported time point.


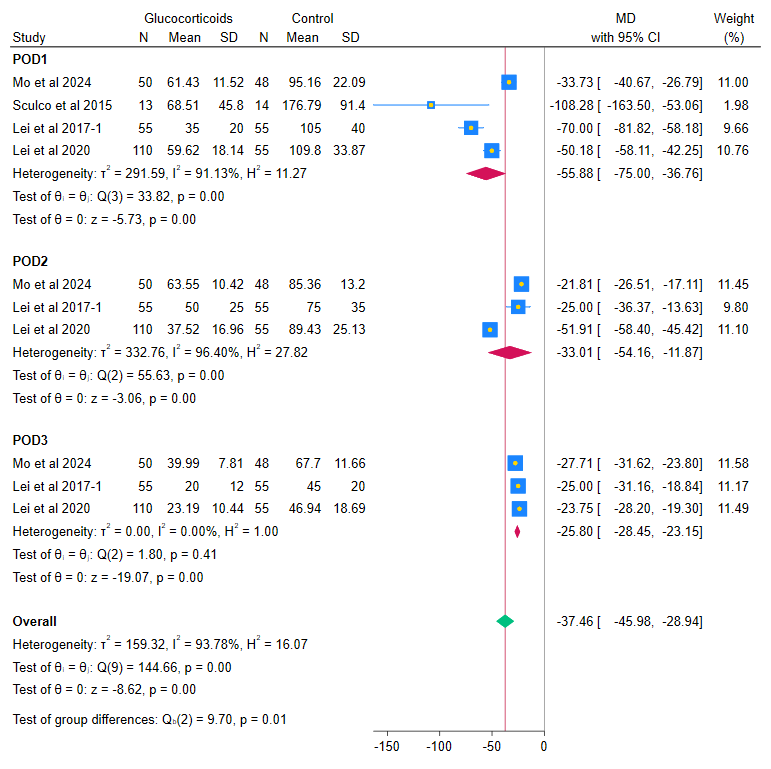


**Supplementary Fig. S5.** Forest plot illustrating the subgroup analysis of Interleukin-6 (IL-6) levels at different follow-up intervals (POD1–3).

**
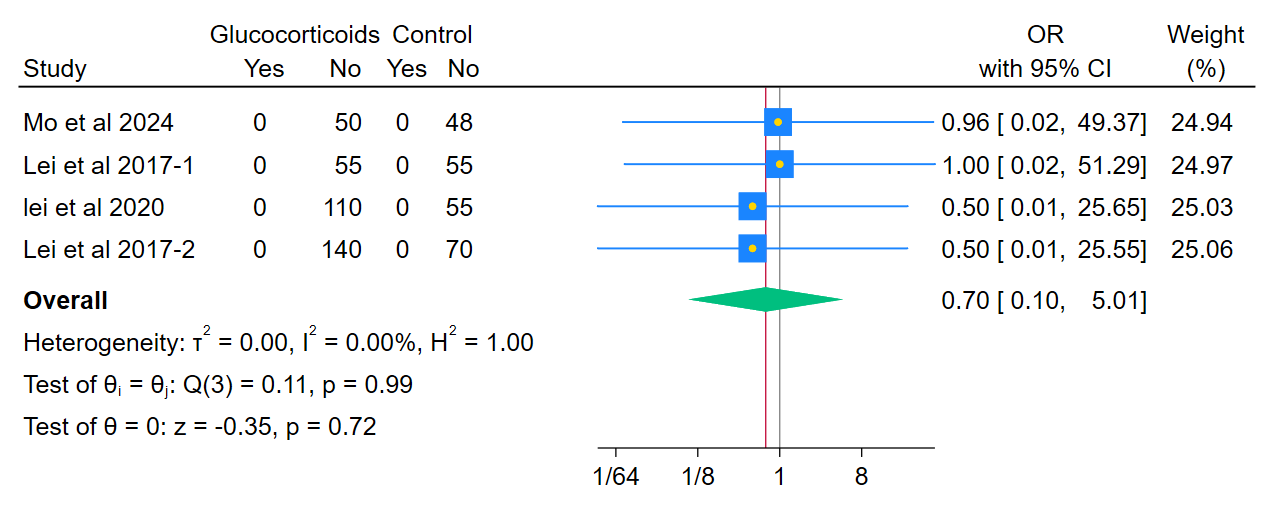
**

**Supplementary Fig. S6.** Forest plot illustrating the incidence of postoperative gastrointestinal bleeding.

**
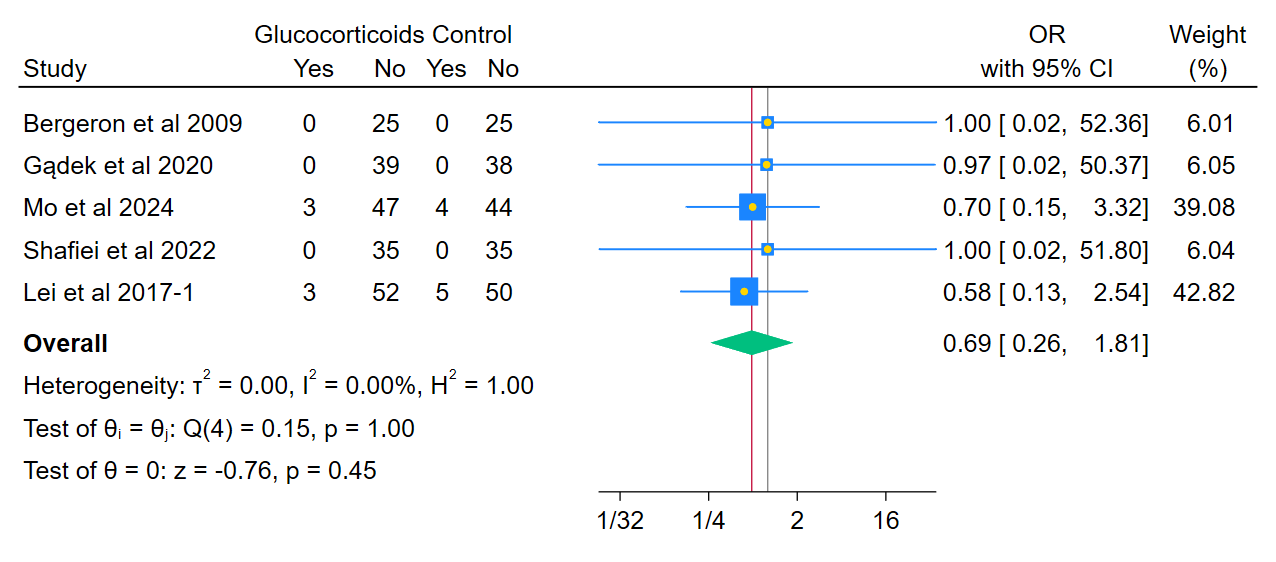
**

**Supplementary Fig. S7.** Forest plot illustrating the incidence of postoperative wound complications.

**
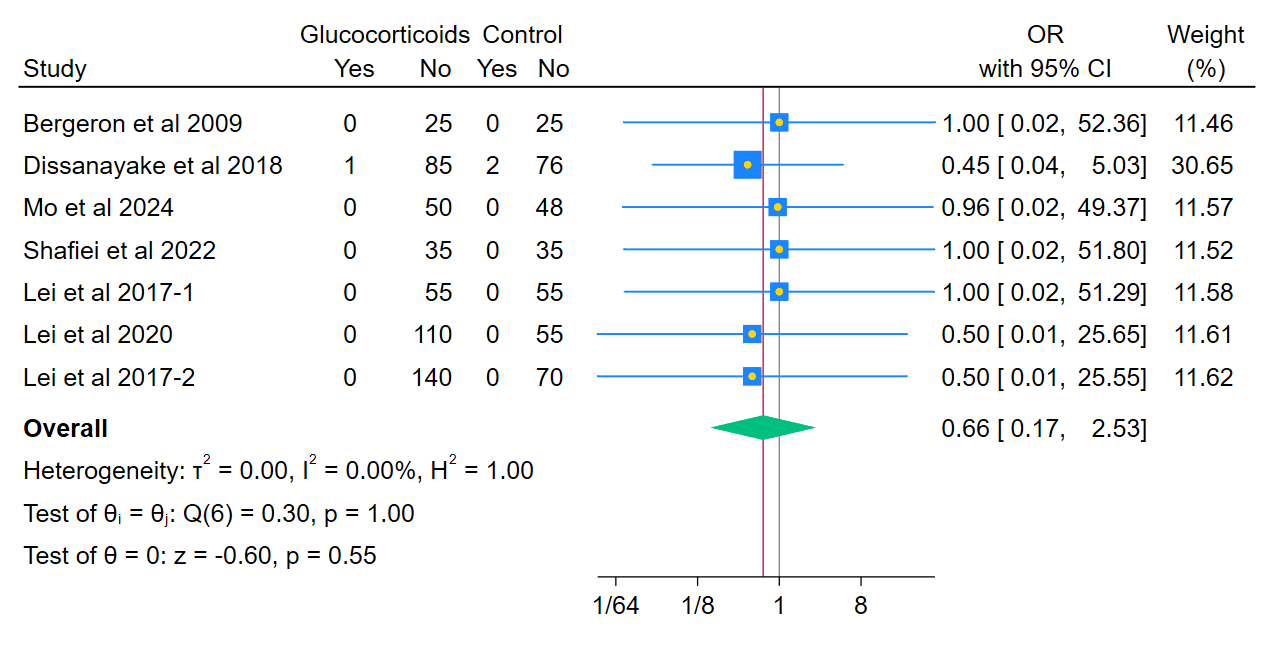
**

**Supplementary Fig. S8.** Forest plot illustrating the incidence of postoperative infection.

**
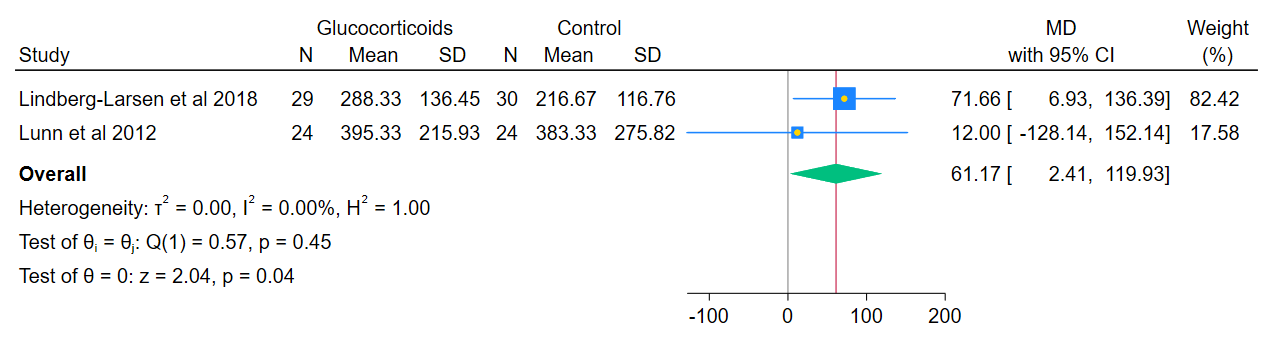
**

**Supplementary Fig. S9.** Forest plot illustrating the difference in intraoperative blood loss.

**
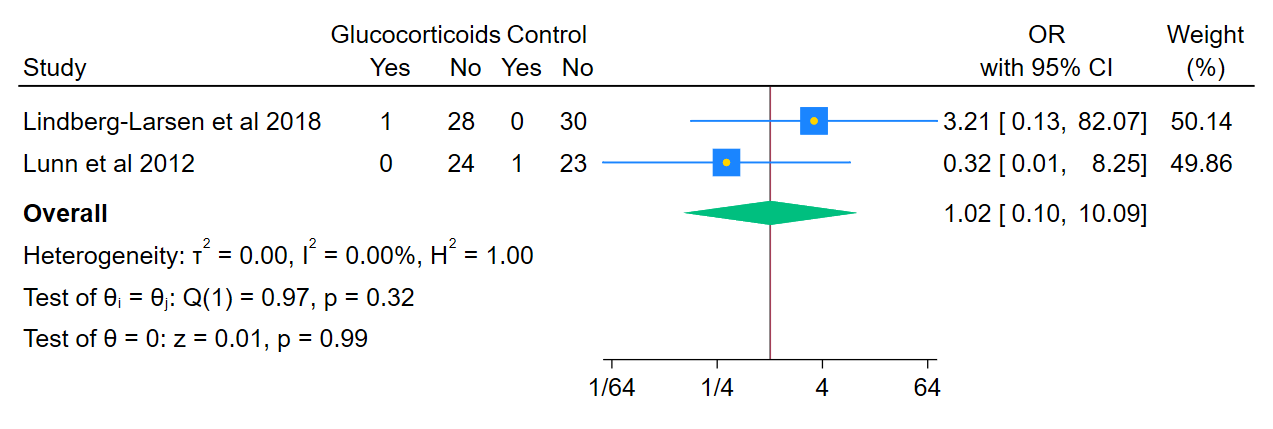
**

**Supplementary Fig. S10.** Forest plot illustrating the incidence of revision procedures.

**
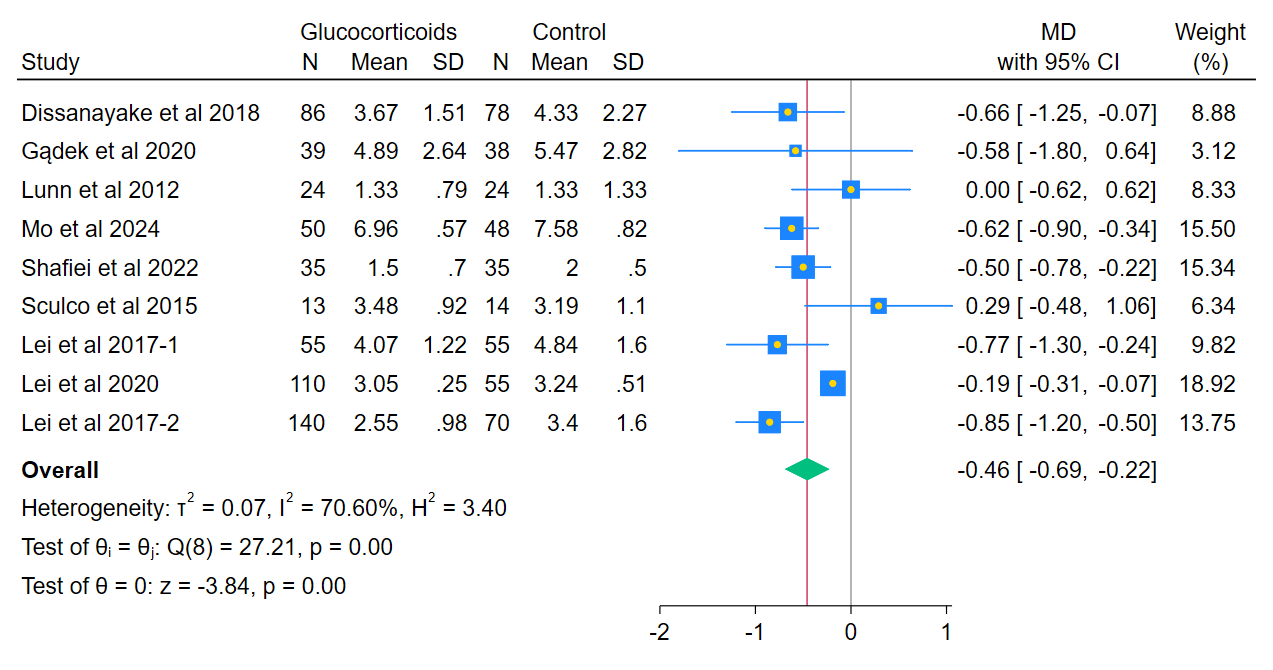
**

**Supplementary Fig. S11.** Forest plot illustrating the mean difference in length of hospital stay (LOS).

**
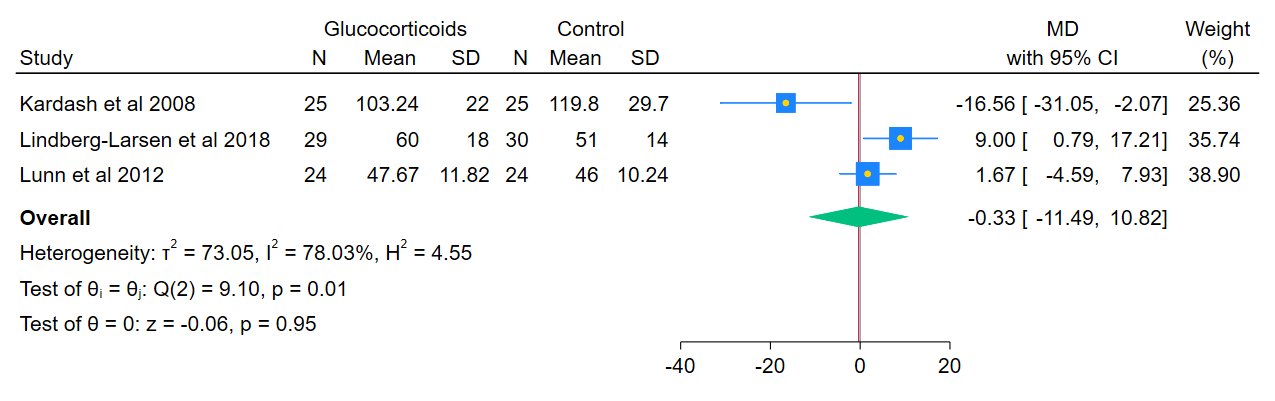
**

**Supplementary Fig. S12.** Forest plot illustrating the difference in operative time.

**
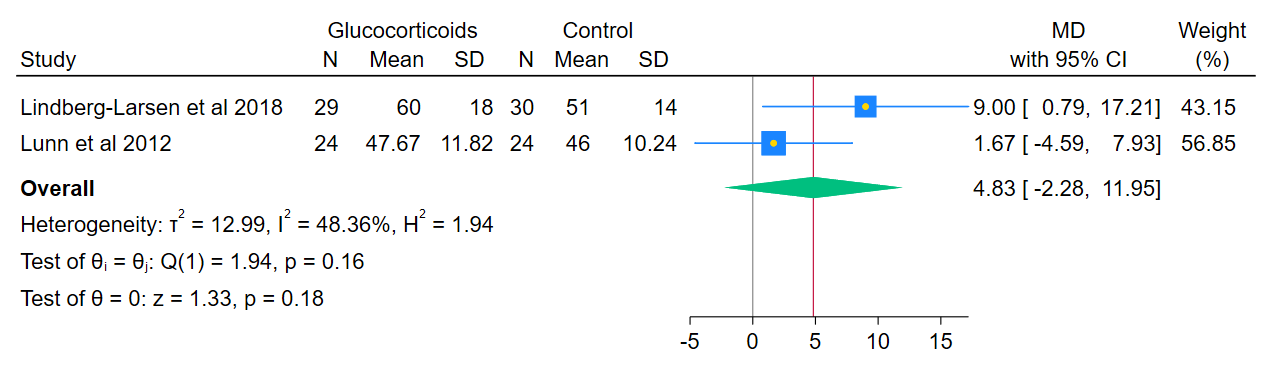
**

**Supplementary Fig. S13.** Forest plot illustrating the difference in operative time after excluding Kardash et al. 2008.

**
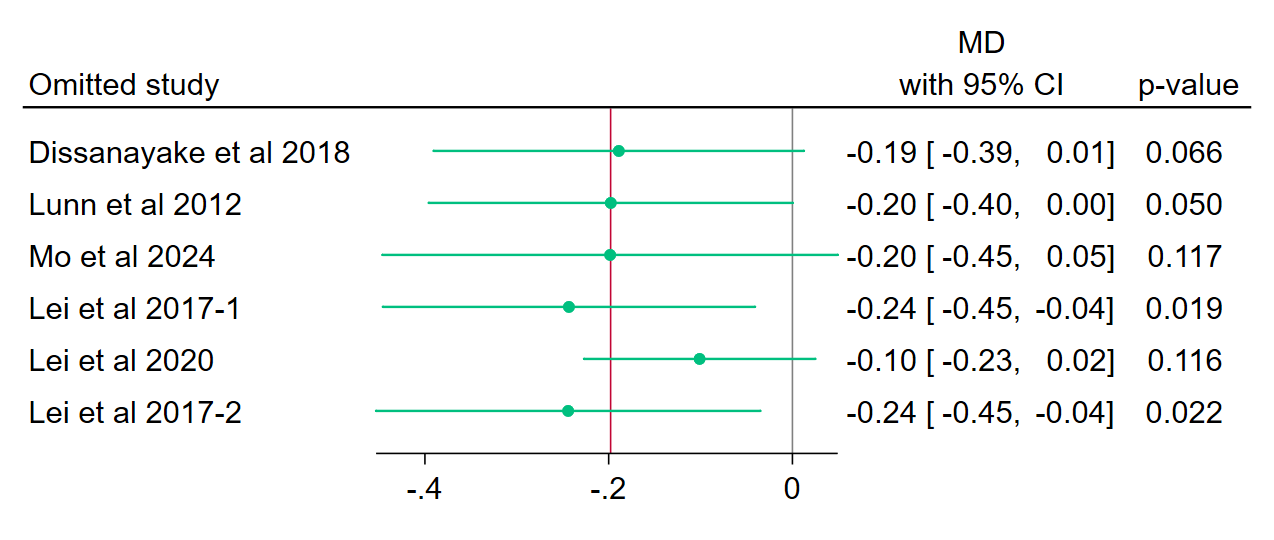
**

**Supplementary Fig. S14.** Leave-one-out analysis for the VAS pain at rest sensitivity analysis outcome, showing the change in the pooled effect after removing each study.

**
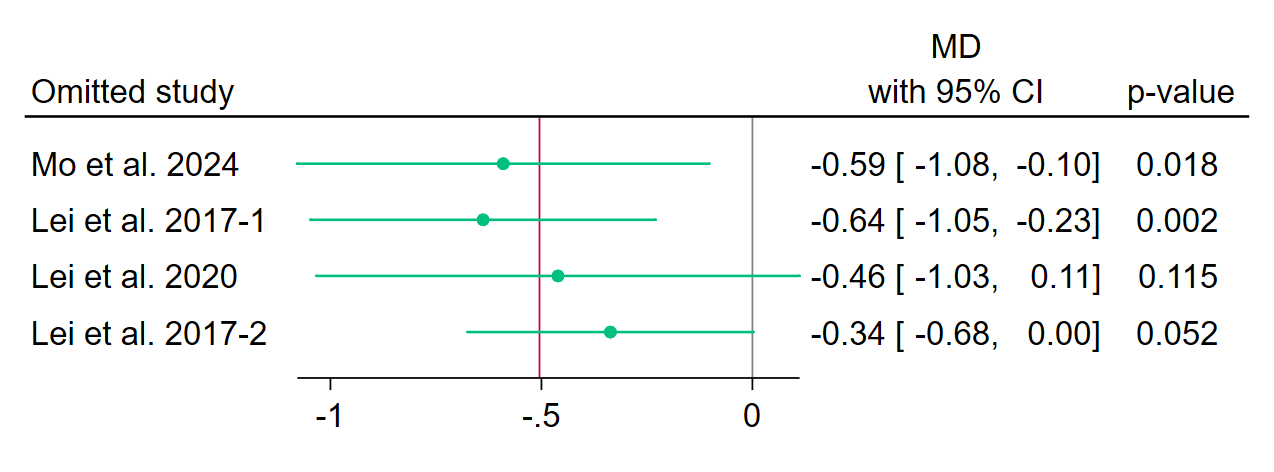
**

**Supplementary Fig. S15.** Leave-one-out analysis for the VAS pain during walk sensitivity analysis outcome, showing the change in the pooled effect after removing each study.

**
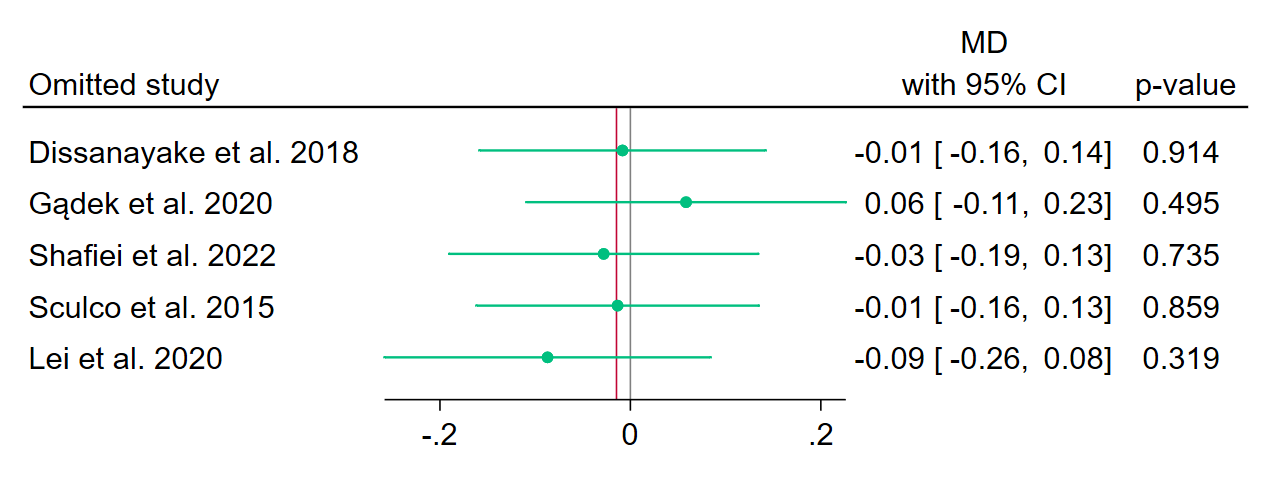
**

**Supplementary Fig. S16.** Leave-one-out analysis for the blood glucose level sensitivity analysis outcome, showing the change in the pooled effect after removing each study.

**
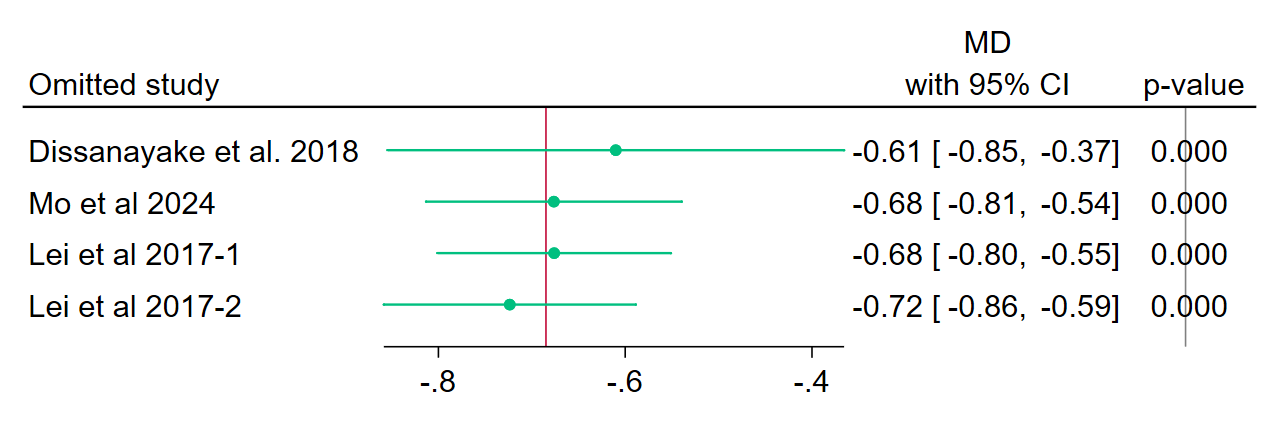
**

**Supplementary Fig. S17.** Leave-one-out analysis for the VAS-Nausea sensitivity analysis outcome, showing the change in the pooled effect after removing each study.

**
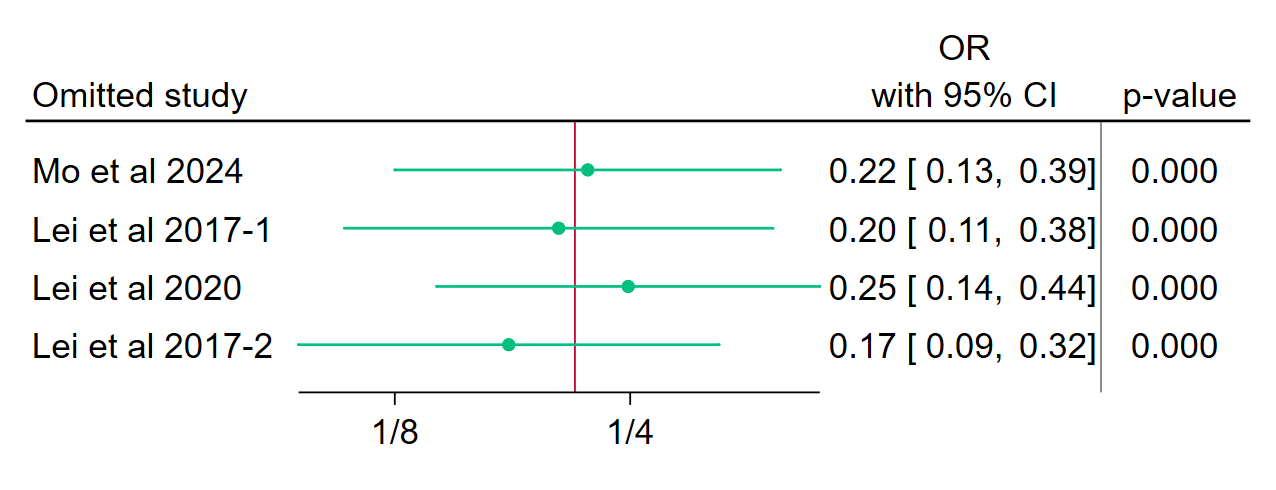
**

**Supplementary Fig. S18.** Leave-one-out analysis for the PONV sensitivity analysis outcome, showing the change in the pooled effect after removing each study.

**
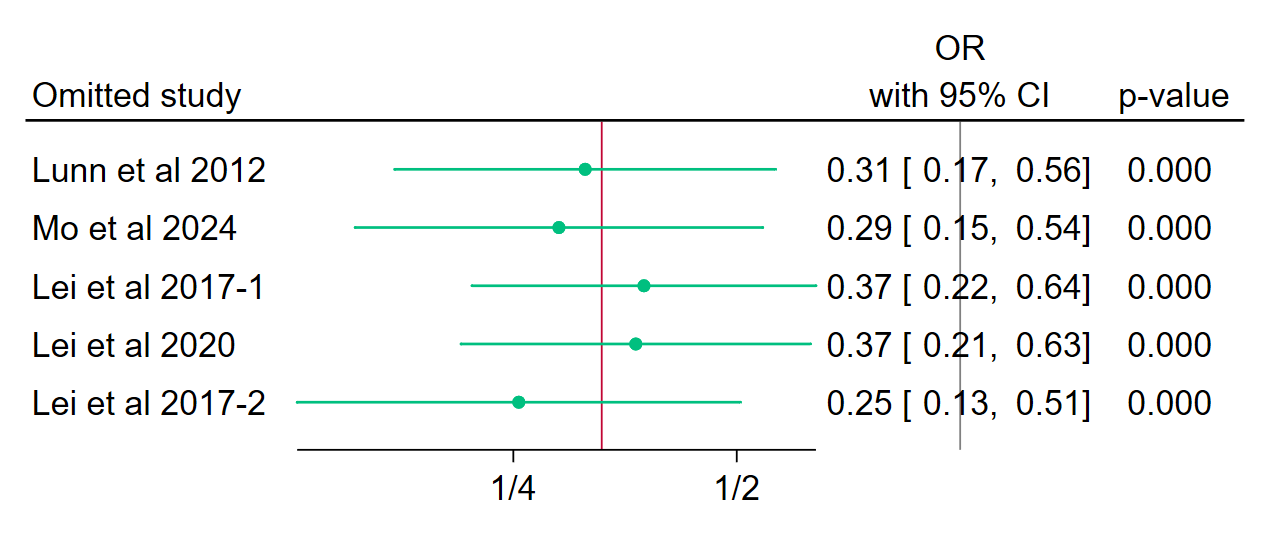
**

**Supplementary Fig. S19.** Leave-one-out analysis for the rescue antiemetic use sensitivity analysis outcome, showing the change in the pooled effect after removing each study**.**

**
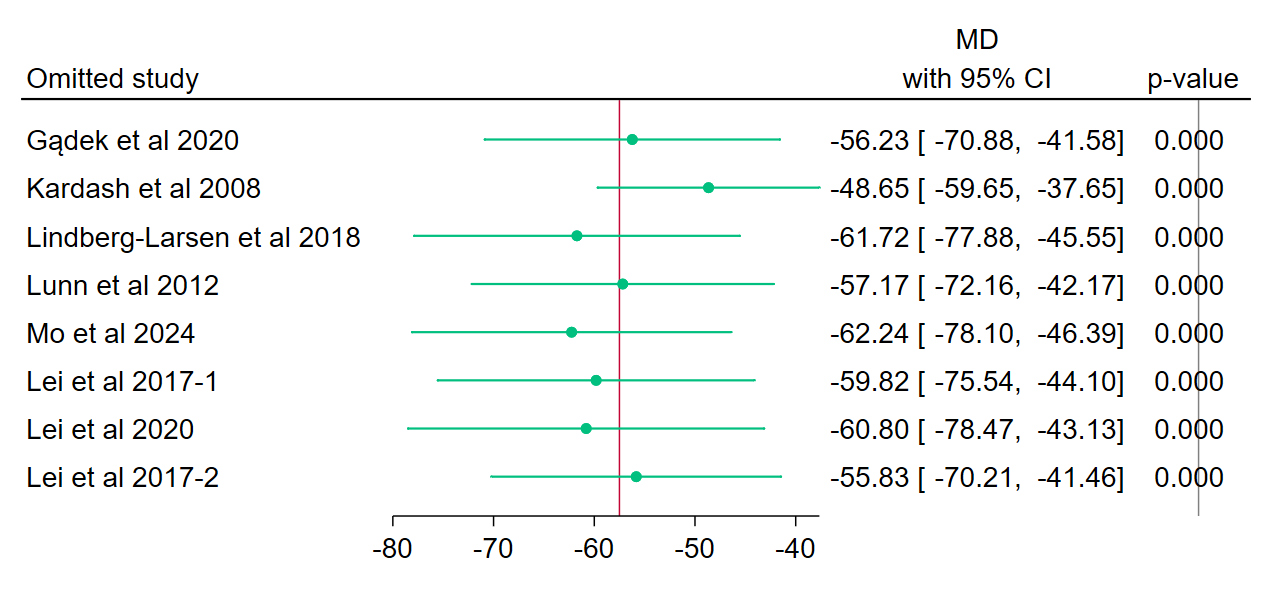
**

**Supplementary Fig. S20.** Leave-one-out analysis for the CRP sensitivity analysis outcome, showing the change in the pooled effect after removing each study.

**
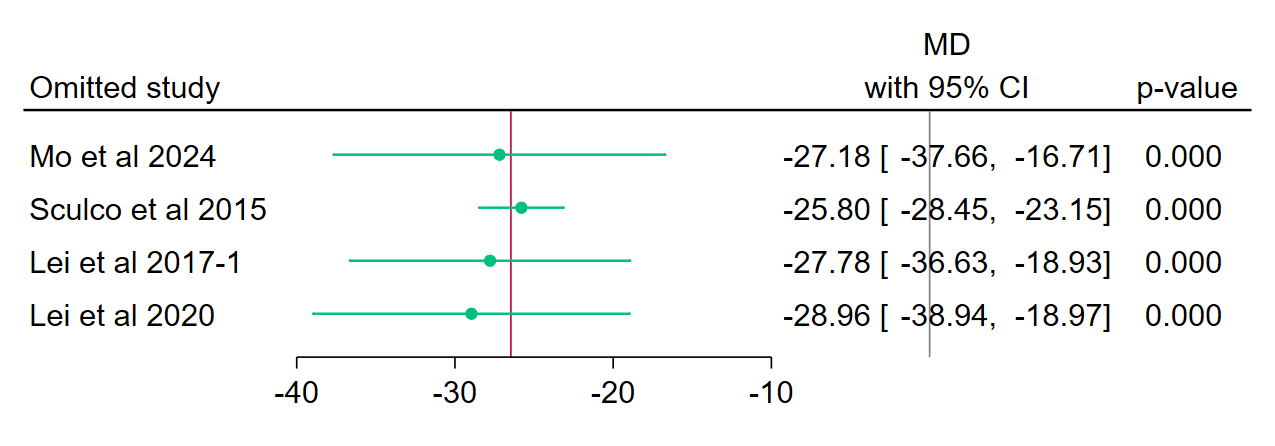
**

**Supplementary Fig. S21.** Leave-one-out analysis for the IL-6 sensitivity analysis outcome, showing the change in the pooled effect after removing each study.

**
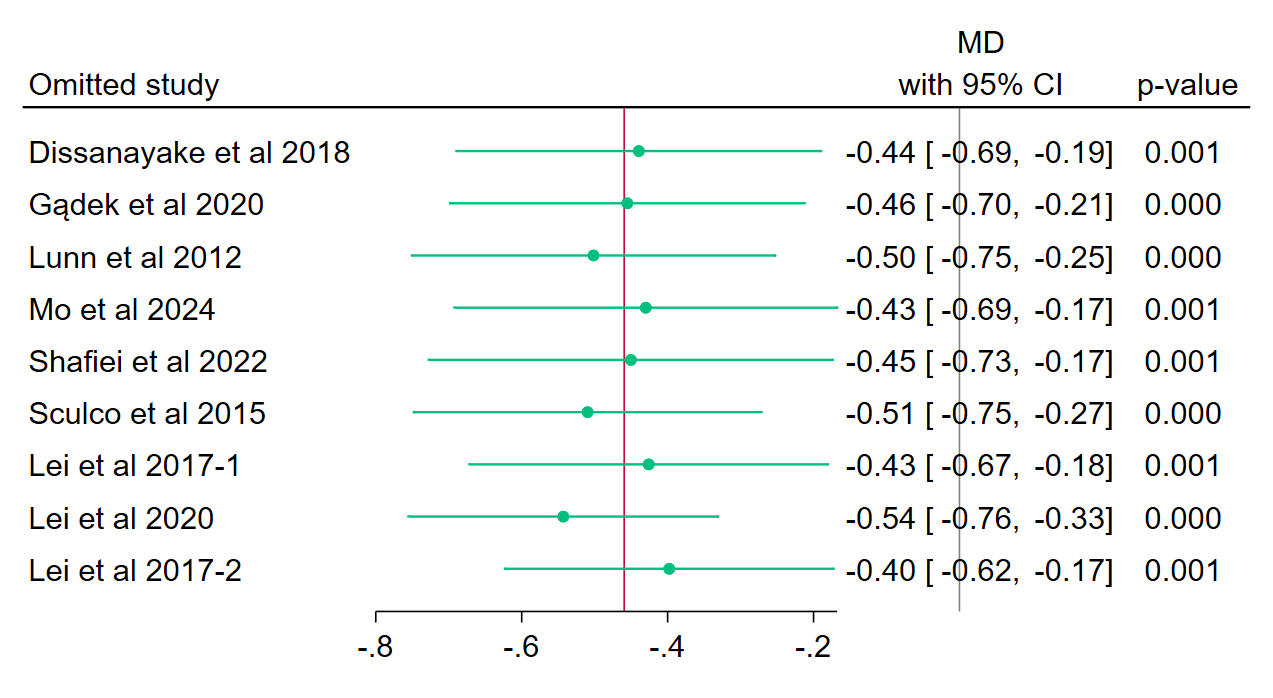
**

**Supplementary Fig. S22.** Leave-one-out analysis for the length of stay sensitivity analysis outcome, showing the change in the pooled effect after removing each study.

**
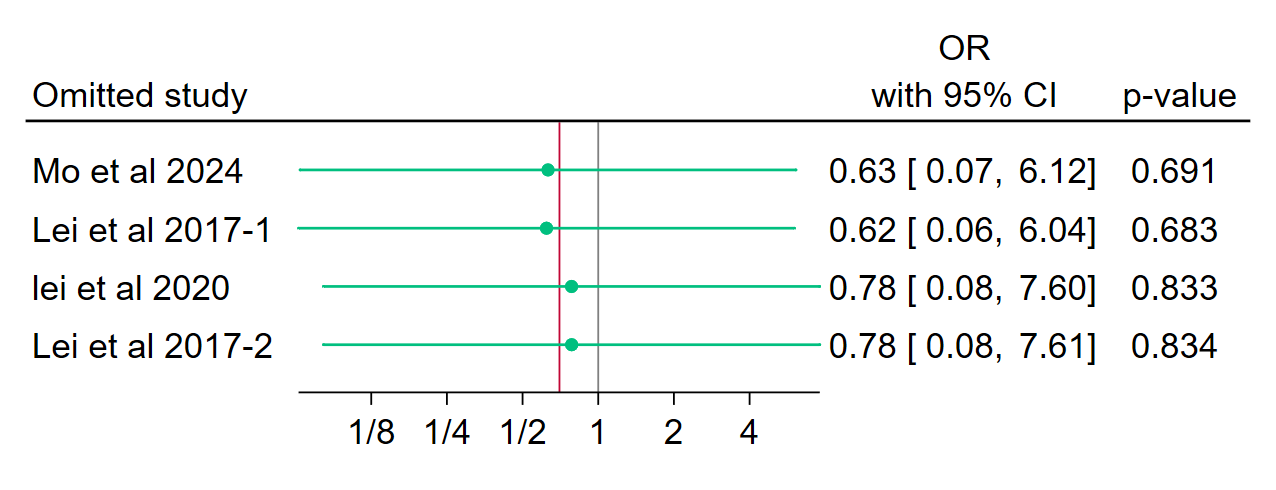
**

**Supplementary Fig. S23.** Leave-one-out analysis for the gastrointestinal bleeding sensitivity analysis outcome, showing the change in the pooled effect after removing each study.

**
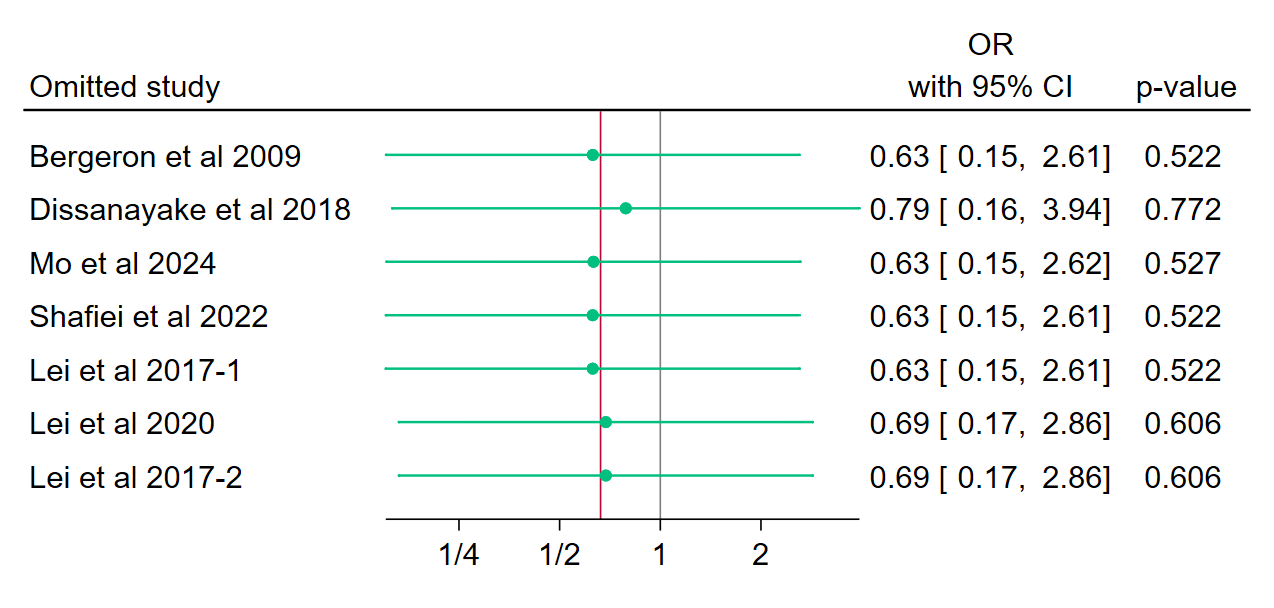
**

**Supplementary Fig. S24.** Leave-one-out analysis for the infection rate sensitivity analysis outcome, showing the change in the pooled effect after removing each study.

**
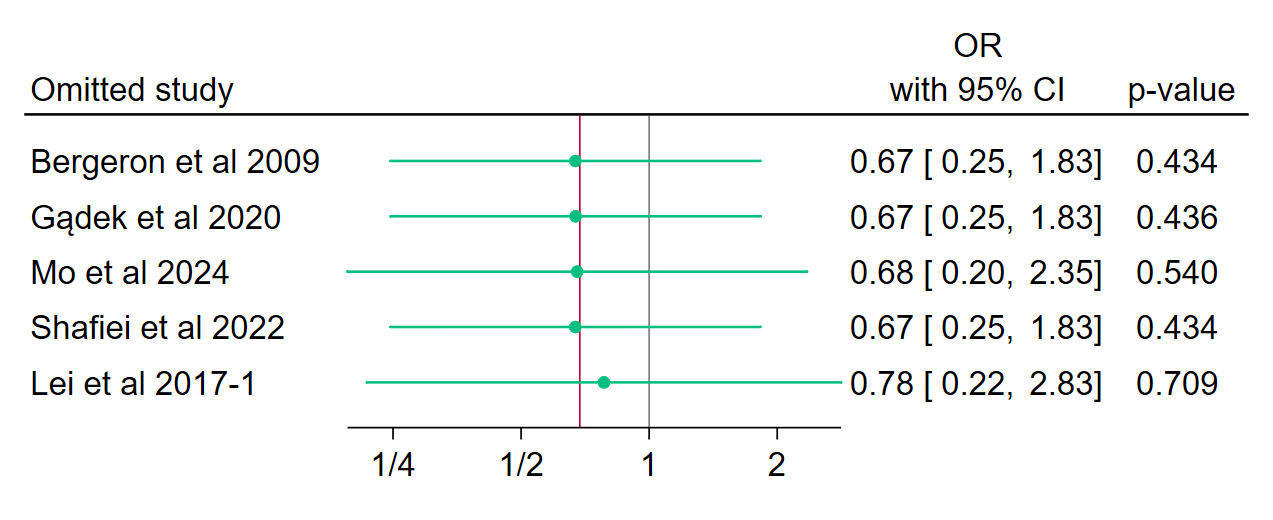
**

**Supplementary Fig. S25.** Leave-one-out analysis for the wound complications’ sensitivity analysis outcome, showing the change in the pooled effect after removing each study.

**
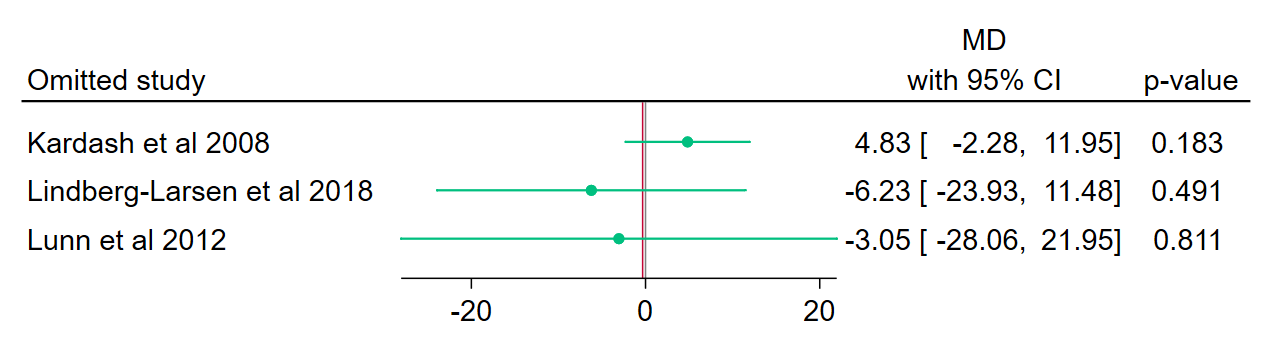
**

**Supplementary Fig. S26.** Leave-one-out analysis for the operative time sensitivity analysis outcome, showing the change in the pooled effect after removing each study.
